# A systemic approach to accident prevention: How control factors influence accident severity and losses across industries

**DOI:** 10.1371/journal.pone.0325393

**Published:** 2025-06-20

**Authors:** Jian Liu, Zhuqing Zhang, Rui Feng

**Affiliations:** 1 School of Resource and Safety Engineering, University of Science and Technology Beijing, Beijing, China; 2 Key Laboratory of High-Efficient Mining and Safety of Metal Mines of the Ministry of Education, University of Science and Technology Beijing, Beijing, China; 3 Research Institute of Macro-Safety Science, University of Science and Technology Beijing, Beijing, China.; University of Wisconsin-Milwaukee, UNITED STATES OF AMERICA

## Abstract

Accidents are often attributed to frontline operator errors, overshadowing higher-level organizational and regulatory factors. This study integrates Systems-Theoretic Accident Model and Processes (STAMP) with fuzzy-set Qualitative Comparative Analysis (fsQCA) and Necessary Condition Analysis (NCA) – a configurational approach – to examine 80 major accident investigation reports from five high-risk Chinese industries (chemical, construction, transportation, coal mining, firefighting) spanning 2010–2022. Four systemic control elements (control activities errors, feedback errors, controller failures, controlled process errors) were assessed against three severity indicators (fatalities, injuries, direct economic losses). Results reveal distinct yet overlapping causal pathways. In chemical accidents, feedback errors are crucial for high fatalities. Construction and coal mining often link early controller/control activity failures to severe outcomes. Transportation highlights control activity errors for injuries, while firefighting points to the combination of control activity errors and controller failures. NCA corroborates key factors like feedback errors and controller failures as necessary conditions (effect sizes d > 0.1, p < 0.05). While supplementary statistical analysis confirmed these factors’ general importance, it faced data limitations (small N, collinearity); the fsQCA/NCA approach provided more robust insights into combinatorial pathways and necessity. Bottleneck analyses further indicate that even modest increments in key errors can trigger disproportionately large losses. These findings underscore the need for multi-level interventions—strengthening feedback loops, organizational oversight, and control processes—to mitigate accident severity in complex socio-technical systems, demonstrating the utility of configurational methods for understanding systemic failures.

## 1. Introduction

Heinrich’s perspective—attributing 88% of accidents to unsafe actions by frontline operators—has profoundly shaped the safety field by emphasizing interventions aimed at mitigating errors at the operational level [1].While this view highlights the importance of individual behavior, some studies stress the need to address broader, systemic factors that can significantly influence accident causality. For instance, Zhou et al. [2] employ binary attributes to annotate human factors in accident reports, whereas La Fata et al. [3] underscore identifying key human elements to improve human reliability in manufacturing. Although these studies acknowledge human factors, they tend to overlook higher-level regulatory or organizational issues that can profoundly affect safety outcomes. Consequently, increasing attention has turned to systems thinking, which expands beyond frontline behavior to consider risk management and accident prevention from a more holistic perspective [4,5]. For example, Li and Wang [6] integrate the Functional Resonance Analysis Method (FRAM) and Multi-Team Systems (MTS) to explore how multi-team coordination influences safety in complex tasks, and Ma and Chen [7] focus on risk identification at the management level, revealing the value of systemic approaches.

Despite the growing application of systems thinking, many analyses still concentrate on lower-level accident factors such as individual behaviors or equipment-related issues [8–11]. Wu et al. [12] developed a hybrid HFACS-SD (Human Factors Analysis Classification System–System Dynamics) model to reveal aviation human factors risk, while Ma et al. [13] identified 60 human factors within HFACS to investigate causal chains for different accident scenarios. Junjia et al. [14] and Zheng et al. [15] similarly highlight human factors but also point out that incomplete data or limited analytical scopes may cause higher-level influences to be underestimated [8]. Notably, regulatory and governmental drivers—though less frequently examined—can exert substantial influence in preventing accidents and mitigating their consequences [16].

To explore these often-overlooked higher-level factors, this study employs the Systems-Theoretic Accident Model and Processes (STAMP) hierarchical safety control structure [17]. STAMP conceptualizes safety as a control problem: systems are seen as dynamic wholes composed of multiple interconnected components, regulated through control and feedback loops [18]. Hence, safety requires managing evolving socio-technical elements [11,17]. Previous work integrating STAMP with methods such as DEMATEL and fuzzy techniques [19], as well as driver distraction analysis [20], underscores the value of examining macro-level elements like regulatory structures, standards, and industry policies. Moreover, the STAMP-Game model [21] and similar system-focused applications [22] further emphasize the interplay of technology, personnel, society, and organizations.

Recognizing the interdependence of control structure factors in socio-technical systems, this study integrates Qualitative Comparative Analysis (QCA) to investigate how multiple combinations of high-level and low-level factors can jointly contribute to accident outcomes [23,24]. We specifically employ Fuzzy-Set QCA (fsQCA) to calibrate and analyze complex causal relationships, thereby illuminating diverse pathways leading to similar accident severities [25]. To complement QCA’s strengths, we incorporate Necessary Condition Analysis (NCA), which quantitatively assesses the necessity of condition variables and their level differences [26,27]. Thus, the combined fsQCA-NCA approach provides a richer, more nuanced understanding of how control factors influence accident severity.

**Fig 1:**
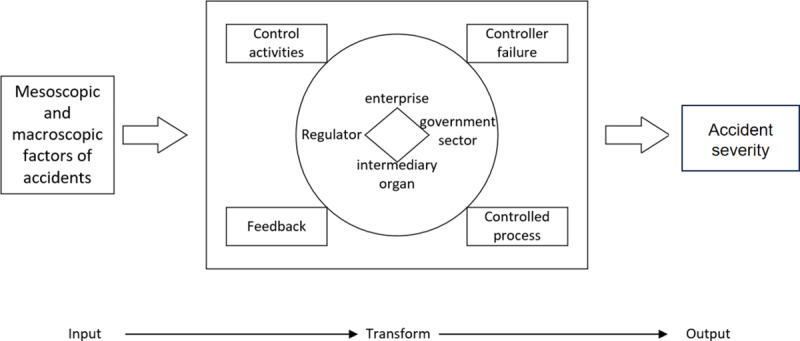
Critical process of methodological flow.

As illustrated in Fig 1, our STAMP-based framework focuses on four interacting control factors and three social system levels [28]: (1) the micro-level (individuals within organizations, such as managers, workers, and experts), (2) the meso-level (organizational factors, including companies, government agencies, and regulatory bodies), and (3) the macro-level (sociological factors, such as laws, policies, and regulations). Guided by these levels, this study aims to reveal how higher-level social system factors shape accident losses and to quantify the influence of each control factor on accident severity.

The remainder of this paper is structured as follows. Section 2: `Analysis of research methods and necessary conditions’ outlines the research methods, including model development, data handling, and the NCA/QCA approaches. Section 3: `Results and discussion’ presents the results and discussion for each industry. Section 4: `Comparative analysis’ provides a comparative analysis of core conditions and accident stages. Section 5: `Conclusion’ concludes with key findings and future research directions.

## 2. Analysis of research methods and necessary conditions

### 2.1. Model development

The concept of control encompasses many safety engineering practices and more. It is important to emphasize that “control” does not involve coercing individuals into compliance with specific roles and protocols through the exertion of power [29]. Both engineering systems and direct management influence it, and it is also subject to indirect control stemming from policies, procedures, values, and organizational culture, his viewpoint is consistent with socio-technical systems theory, which emphasizes the interaction between technical systems and social systems [30].

The STAMP model, built on three foundational concepts – safety constraints, hierarchical safety control structures, and process models – underscores the crucial role of safety constraints. Leveson [17] points out that safety issues are defined as control problems for achieving constraints, a viewpoint derived from systems control theory [31]. The importance of safety constraints is particularly emphasized in the STAMP model [32].

Hierarchical control structures represent system models comprising multiple feedback control loops. Within these structures, controllers manage specific processes by implementing control activities, which, in turn, impose constraints on the behaviors of those processes. Since most systems involve multiple, intertwined control loops, hierarchical control structures become indispensable for modeling these complexities, this hierarchical structure draws on the ideas of cybernetics and systems theory [33]. Control activities permeate every layer of the structure, descending from higher echelons to the lower levels and ascending as feedback from the base to the apex [34].

**Fig 2:**
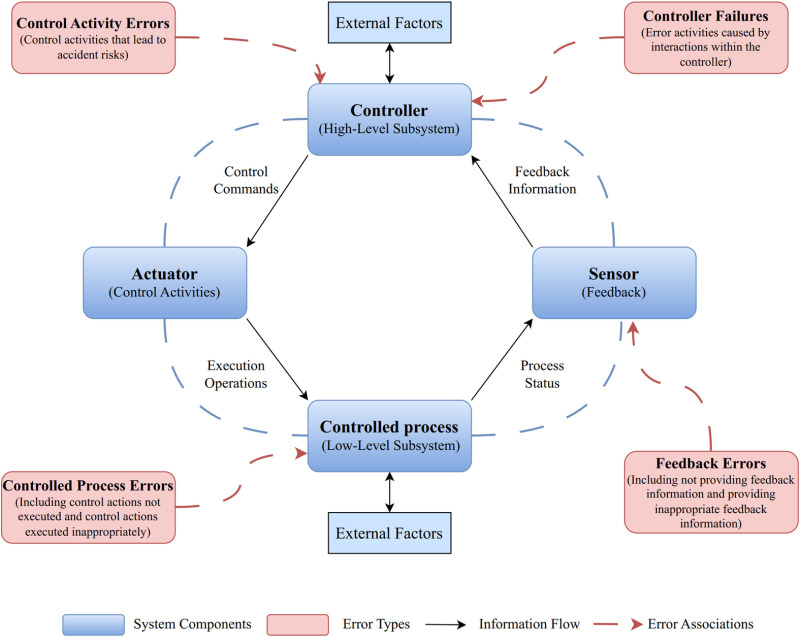
Definition of control factors based on STAMP control structure.

The process model is a core component of the STAMP model, requiring a control model to effectively manage it, whether the processes are manual or automated. In a specific system, system constraints are enforced by controllers through the behavior and interactions between components [32]. Each controller not only implements constraints but is also constrained by other controllers. In social systems, individual factors at the micro-level are influenced by organizational factors at the meso-level and societal factors at the macro-level [35]. Fig 2 provides a visual representation of a hierarchical control structure, illustrating control loops involving upper and lower tiers and the interconnected control and feedback processes.

Each organization mentioned in the accident investigation report is considered a controller, and these controllers are the most minor units of study. The controller transfers control activities to the controlled process through the actuator. The organization’s feedback is mainly based on audits, reports, and incident and accident analyses.

Controllers implement system constraints in specific systems through interactions and behaviors among components. Each controller is responsible for enforcing controls while subjecting to the limitations imposed by other controllers. Within social systems, individual factors at the micro-level are influenced by organizational factors at the meso-level and societal factors at the macro-level [35].

**Fig 3:**
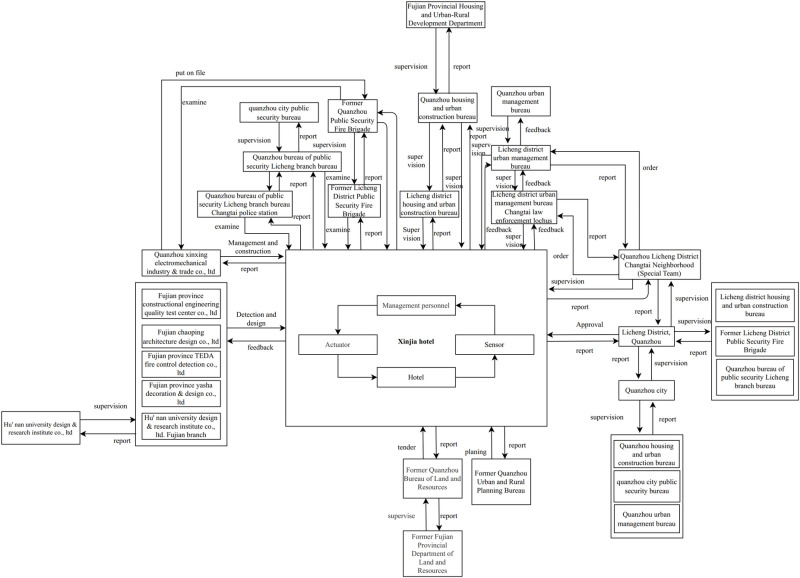
(Please see the attached high-resolution image) Control structure diagram of the Xinjia Hotel collapse accident.

Consider the example of the “3·7” collapse accident at Xinjia Hotel in Quanzhou City, Fujian Province, as illustrated in Fig 3 [36]. In this accident, Xinjia Hotel, the establishment where the accident occurred, holds a central position. Organizations directly linked to it, such as the Licang District Housing and Urban-Rural Construction Bureau, maintain direct control and feedback relationships. Xinjia Hotel is obliged to relay information to these regulatory entities promptly. On the other hand, Quanzhou City Housing and Urban-Rural Construction Bureau, as a higher-level department, assumes supervisory responsibilities over Xinjia Hotel across various levels.

Within this control structure, the controlled processes of Xinjia Hotel involve implementing control activities imposed by higher-level organizations. If internal organizational factors lead to erroneous actions, these are controller failures. This study encompasses enterprises where accidents occur, related enterprises, intermediary agencies, regulatory bodies, and government departments. It conducts a comprehensive analysis from the vantage points of control activities, feedback, controlled processes, and controller failures.

The primary entity involved in the accident is the Xinjia Hotel, with associated organizations including intermediary agencies (such as the Hunan University Design and Research Institute Co., Ltd.), regulatory bodies (such as the Licheng District Housing and Urban-Rural Development Bureau), and local government (such as Licheng District, Quanzhou City). All connected organizations have control structures.

### 2.2. Research method

Qualitative Comparative Analysis (QCA) is rooted in Boolean algebra and focuses on examining antecedent conditions in multiple case samples to reveal combinations of factors that lead to specific outcome variables. It highlights the intricate and diverse causal relationships between antecedent conditions and outcome variables [37]. QCA’s capacity to capture multiple equifinal pathways makes it particularly valuable for investigating managerial and organizational phenomena in complex contexts [38,39]. Fuzzy-set Qualitative Comparative Analysis (fsQCA), a derivative of QCA, provides detailed insights into these complex causal dynamics by quantifying the membership scores of each antecedent condition [40].

In contrast, Necessary Condition Analysis (NCA) delves into the essential and adequate conditions that connect antecedent states to outcome variables [26]. In NCA, a condition is considered necessary if its presence is indispensable for manifesting the outcome variable. This methodology, suitable for continuous and discrete variables, quantifies the degree of indispensability of each antecedent condition concerning the outcome variable. It plays a crucial role in confirming the intrinsic connection between an outcome variable and specific antecedent requirements while quantifying the extent of influence exerted by these necessary conditions.

The combined use of fsQCA and NCA enhances the robustness and granularity of the research findings, thereby augmenting their intrinsic value. The consistency of the necessity fuzzy subset relationship is evaluated as:


$$Consistency(Y_{i}\leq X_{i}=\sum min(X_{i},Y_{i})/\sum \left(X_{i}\right).$$ (1)

Equation (2) is used to evaluate the coverage of antecedent condition combinations on the outcome variable.


$$Consistency(Y_{i}\leq X_{i}=\sum min(X_{i},Y_{i})/\sum \left(Y_{i}\right).$$ (2)

We first collected variable data from accident reports and used SPSS for descriptive analysis to observe the correlations between various antecedent variables. Furthermore, based on the identified condition variables and outcome variables, we used fsQCA for calibration, converting variable values from interval scale to fuzzy membership scores. To determine which factors are necessary conditions for the severity of accidents and their degree of necessity under a specific control perspective, this study utilized the NCA package in R software. This analysis helped us to better understand the causal dynamics under different control structures. Additionally, we verified the robustness of these necessary conditions through fsQCA’s single condition analysis, thereby identifying multiple configuration paths that influence the severity of accidents. Finally, fuzzy set analysis was conducted in fsQCA software to construct the driving paths of the outcome variables, thoroughly exploring the necessary and sufficient causal relationships between different variables in the control loop and accident losses.

### 2.3. Research data

#### 2.3.1. Data selection.

The fundamental objective of the fsQCA method is to uncover diverse combinations of conditions that result in specific outcomes. To achieve this goal, the study adopted a deliberate approach to selecting cases to maximize variability across relevant conditions and outcomes, facilitating the identification of a wide range of causal pathways. In the context of China, accident severity was assessed using three metrics: death toll (DT), injuries (IN), and the extent of direct economic losses (EL).

We focused our research on the chemical, construction, transportation, coal mining, and fire protection sectors. These industries were selected due to their recognized high-risk profiles, their significant contribution to severe accident statistics in China, and their representation of diverse complex socio-technical systems suitable for investigating systemic control failures via the STAMP framework. Utilizing official accident investigation reports (format in S1 Appendix), we compiled a dataset of 80 cases spanning 2010–2022. The detailed information about these 80 accident cases is provided in S2 Dataset.

This timeframe was necessary to gather sufficient severe and extremely severe accident cases (N = 80) across these five industries for robust QCA. Within this period, we deliberately prioritized the most comprehensively documented reports, essential for the detailed systemic analysis required by STAMP. While acknowledging potential temporal variations over 12 years, the focus on fundamental control flaws (STAMP) and QCA’s ability to identify consistent configurations across diverse settings support the relevance of this carefully curated dataset for understanding pathways to accident severity.

The final set of selected cases exhibits diversity in terms of region, accident type, company size, and severity levels, making them highly suitable for QCA. After collating and examining these high-quality reports, errors related to control activities, feedback, controller function, and the controlled process within implicated organizations were systematically documented based on the STAMP framework. These data were then analyzed against the severity metrics (DT, IN, EL) to identify configurations of factors potentially modulating accident severity.

#### 2.3.2. Data calibration.

In fsQCA, data preprocessing involves converting the values of condition and outcome variables from interval scale values into fuzzy membership scores that range from 0.0 to 1.0. Data preprocessing is crucial in creating fuzzy sets and provides richer information than uncalibrated values [23].

The “calibration” feature of fsQCA 3.0 software was utilized for this purpose. An indirect approach was used to process the cause and result variables, involving qualitative assessments by researchers to determine the membership degree of specific scores within a designated set. To construct a fuzzy set, it was necessary to identify three qualitative breakpoints: full membership (fuzzy score 0.90), full non-membership (fuzzy score 0.10), and the crossover point (fuzzy score 0.50) [41]. A constant of 0.001 was added to the 0.50 membership score to handle cases where antecedent conditions were precisely at 0.50 [42]. Leveraging these thresholds, the software transformed raw data into calibrated fuzzy membership scores, encompassing death toll, injuries, direct economic losses, control activities errors, feedback errors, controlled processes errors, and controller failures. After this calibration, the analysis primarily focused on identifying diverse combinations of causal conditions that culminate in specific result memberships, as shown in Table 1, full membership indicates that a case completely possesses a certain attribute, the crossover point represents a neutral state regarding the attribute, and full non-membership indicates that a case entirely lacks the attribute.

**Table 1:** Calibration anchors of each fuzzy set.

Type	Outcome/conditions	Calibration		
Full membership	Crossover point	Full non-membership
Chemical	Control activities errors	55.40	10.00	2.70
	Feedback errors	3.60	1.00	0.00
	Controller failure	30.30	13.00	6.40
	Controlled process errors	5.60	1.00	0.00
	Death toll	104.10	10.00	3.00
	Injuries	334.60	10.50	0.70
	Direct economic loss	345024.55	4145.43	514.75
Transportation	Control activities errors	22.80	10.00	3.00
	Feedback errors	3.60	1.50	0.00
	Controller failure	24.80	13.00	4.80
	Controlled process errors	4.80	1.00	0.00
	Death toll	37.20	16.60	4.40
	Injuries	32.50	9.00	0.00
	Direct economic loss	7429.10	2461.40	356.47
Construction	Control activities errors	38.60	6.50	2.70
	Feedback errors	3.30	1.00	0.00
	Controller failure	30.90	9.00	2.00
	Controlled process errors	5.00	0.10	0.00
	Death toll	73.00	10.50	3.00
	Injuries	24.50	2.50	0.00
	Direct economic loss	9227.72	1089.30	196.00
Coal mining	Control activities errors	29.80	8.00	5.70
	Feedback errors	2.00	1.00	0.00
	Controller failure	21.00	14.50	9.40
	Controlled process errors	3.90	1.00	0.00
	Death toll	32.30	15.00	3.70
	Injuries	26.60	1.00	0.00
	Direct economic loss	6068.02	2406.00	852.76
Firefighting	Control activities errors	22.40	10.00	3.70
	Feedback errors	1.30	1.00	0.00
	Controller failure	25.60	10.00	03.70
	Controlled process errors	1.00	0.10	0.00
	Death toll	63.60	15.50	2.80
	Injuries	38.90	5.00	0.70
	Direct economic loss	11721.46	1490.60	0.00

An important aspect to highlight is the emphasis on analyzing combinations of conditions rather than the isolated net effects of individual causal needs. QCA emphasizes that various combinations of situations can lead to identical outcomes, while similar combinations can result in different results. Using fsQCA 3.0 software allowed for identifying these combinations’ necessary and sufficient conditions, facilitating a more intricate analysis.

### 2.4. Necessary condition analysis

#### 2.4.1. Analysis of necessary conditions of NCA.

**Fig 4:**
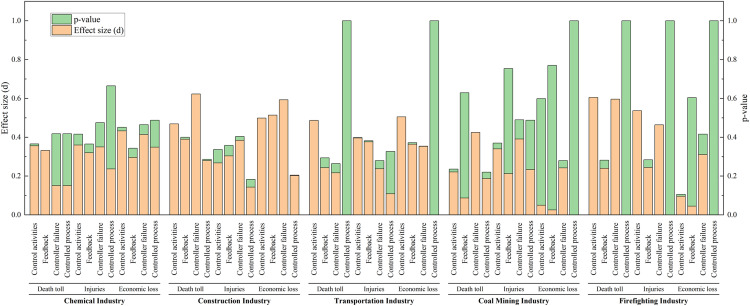
NCA necessary condition evaluation.

In the context of Necessary Condition Analysis (NCA), a variable is classified as a necessary condition if it meets at least three criteria: (1) it possesses theoretical relevance for the outcome, (2) it demonstrates a non-negligible effect size (conventionally, d > 0.1), and (3) the necessity finding is statistically significant (typically, p < 0.05 via permutation tests). This study utilized the NCA package within the R environment to analyze the calibrated fuzzy-set membership scores. The results, including effect sizes and significance levels determining which control factors qualify as necessary conditions according to NCA methodology across different industries, are presented in Fig 4. Furthermore, since the degree of necessity is often not uniform across the range of the outcome, a bottleneck-level analysis was conducted within NCA. This analysis identifies the minimum level (threshold) of a necessary condition required to achieve a specific level of the outcome, offering a more granular understanding of the constraint imposed by the necessary condition.

#### 2.4.2. Qualitative comparative analysis (QCA): necessity and sufficiency.

**Fig 5:**
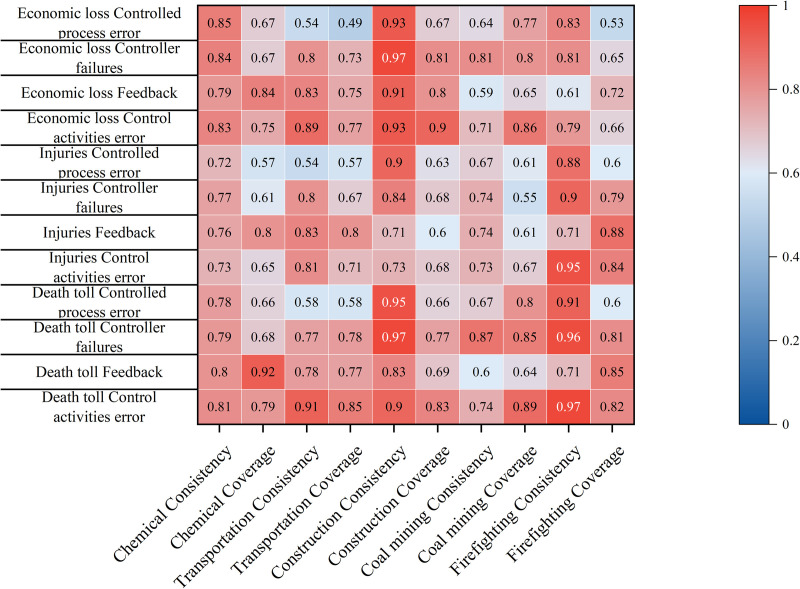
QCA necessary condition evaluation.

Within the framework of Qualitative Comparative Analysis (QCA), assessing necessity is also a standard preliminary step before proceeding to the core analysis of sufficient condition combinations [24]. In QCA terms, a condition is considered necessary if it is consistently present whenever the outcome occurs (though its presence alone may not guarantee the outcome). This initial step aims to identify if any individual causal factors act as prerequisites for high accident losses in the dataset. For this study, following common QCA practice [43], a consistency score threshold of ≥0.9 was used to identify such necessary conditions within the QCA framework. Conditions meeting this criterion are highlighted as potentially necessary prerequisites for the outcome. The results of this QCA-based necessity assessment are depicted in Fig 5.

Following the initial necessity assessment and fuzzy-set calibration of all conditions, a truth table was constructed. This table systematically lists all logically possible combinations of the four causal conditions (2^k^ = 2^4^ = 16 combinations) and summarizes the empirical evidence associated with each combination based on the dataset. Each row represents a unique configuration of conditions linked to the outcome (high accident losses). This truth table serves as the foundation for the subsequent sufficiency analysis.

The primary objective of sufficiency analysis in QCA is to identify specific configurations (combinations or pathways) of causal conditions that consistently and reliably lead to the outcome of interest (in this case, high accident losses). Unlike necessity analysis, which checks if single conditions must be present, sufficiency analysis determines which sets of conditions are jointly adequate to produce the outcome, acknowledging that multiple distinct pathways (equifinality) may exist.

To empirically determine which configurations derived from the truth table represent sufficient pathways to the outcome, specific analytic benchmarks were applied. In this study, a consistency threshold of ≥0.80, a minimum case frequency threshold of 1, and a Proportional Reduction in Inconsistency (PRI) score benchmark of ≥0.50 [44] were employed. These criteria ensure that only configurations consistently associated with the outcome, supported by at least one empirical case, and not simultaneously strongly linked to the absence of the outcome are retained for interpretation.

The fsQCA software then generates three types of solutions based on differing assumptions about logical remainders: the complex solution (incorporating no simplifying assumptions), the parsimonious solution (incorporating all possible simplifying assumptions consistent with theory and evidence), and the intermediate solution (incorporating only those simplifying assumptions deemed theoretically or empirically plausible by the researcher) [25]. Following established QCA guidelines, causal conditions that are part of both the parsimonious and intermediate solutions are identified as core conditions, suggesting a strong and central causal relationship. Conversely, conditions that appear only in the intermediate solution (but are absent from the parsimonious solution due to sim.

Recognizing that QCA involves several analytical decisions [45], robustness checks were performed, specifically focusing on the stability of the sufficiency analysis results. By systematically altering the parameters for sufficiency analysis (e.g., adjusting the consistency threshold for sufficiency to 0.85, increasing the case frequency threshold to 2, and raising the PRI benchmark to ≥0.60), the stability of the identified sufficient configurations was assessed. These checks yielded results largely congruent with the primary analysis. While minor variations in consistency or coverage scores, or the classification of some conditions as core versus peripheral, were observed across different parameter settings, no discrepancies were substantial enough to necessitate a fundamentally different interpretation of the causal pathways leading to high accident losses. Consequently, the findings related to the sufficient configurations are considered robust and reliable.

## 3. Results and discussion

In this section, the NCA bottleneck level analysis reveals the percentile level of the condition variable required to attain a certain percentile level of the result variable. Since the CR method is more suitable for practical bottleneck-level research [46], this study systematically reports the results of the NCA bottleneck analysis using the CR method based on the necessary condition analysis. The results indicate that the bottleneck levels of the condition variables vary for different levels of the result variables. In the table, “NN” suggests that the condition is unnecessary.

The fsQCA analysis results are represented using the mainstream method [47]. In the representation:

- “⬤” indicates the presence of a core condition,
- “⨂” indicates the absence of a condition,
- “●” indicates the presence of a peripheral condition,
- “⊗” indicates the absence of a peripheral condition,
- a blank space indicates that the condition can be present or absent.

Table 2 illustrates the combinations found within intermediate and parsimonious solutions, differentiating between core and peripheral conditions unique to the intermediate solution. The table also presents all solution pathways, along with the consistency and coverage of the overall solution. Each configuration resulting from the QCA analysis is numbered, as shown in Table 2. This structured approach enhances the clarity and precision of presenting intricate analysis results.

**Table 2:** Configuration numbers from QCA analysis for various industries.

Industry	Loss type	Number
Chemical	Death toll	CD
	Injuries	CI
	Economic loss	CE
Construction	Death toll	BD
	Injuries	BI
	Economic loss	BE
Transportation	Death toll	TD
	Injuries	TI
	Economic loss	TE
Coal mining	Death toll	MD
	Injuries	MI
	Economic loss	ME
Firefighting	Death toll	FD
	Injuries	FI
	Economic loss	FE

### 3.1. Chemical industry

Table 3 presents the QCA results for accident severity in the chemical industry. For high fatalities, configurations CD1 and CD2 both highlight feedback errors as a core condition; CD2 further requires the absence of controlled process errors. For high injuries, CI1 and CI2 likewise center on feedback errors—CI1 specifies their presence and the absence of controlled process errors as core, whereas CI2 only requires the presence of feedback errors. For high economic loss, CE1 includes control activity errors, feedback errors, and controller failures as core, while CE2 combines feedback errors with the presence of controlled process errors.

**Table 3:** Chemical industry high severity accident development and non-high severity accident development conditions.

Configuration	High severity of accident development						Non-high severity of accident development								
Death toll		Injuries		Economic loss		~Death toll		~Injuries				~Economic loss		
CD1	CD2	CI1	CI2	CE1	CE2	N-CD1	N-CD2	N-CI1	N-CI2	N-CI3	N-CE1		N-CE2	N-CE3
Control activities error	⊗	●	⊗	●	●	⊗	⊗	●	⊗	⊗	●	⨂		⊗	●
Feedback error	⬤	⬤	⬤	⬤	⬤	⬤	⨂	⨂	⨂		⨂			⨂	⨂
Controller failure	⊗	●	⊗	●	●	⊗	⊗	●	⊗	⨂	●	⨂		⊗	●
Controlled process error			⨂	⨂		⬤		⊗		⬤	⊗	⨂			⊗
Consistency	1	0.926	0.983	0.982	0.912	1	0.932	0.967	0.906	0.929	0.962	0.981		0.923	0.962
Raw coverage	0.345	0.676	0.283	0.260	0.716	0.257	0.708	0.195	0.652	0.298	0.184	0.598		0.667	0.185
Unique coverage	0.083	0.414	0.065	0.041	0.492	0.033	0.523	0.009	0.288	0.003	0.009	0.045		0.114	0.009
Solution consistency	0.934		0.985		0.916		0.928		0.889				0.914		
Solution coverage	0.759		0.324		0.749		0.718		0.665				0.722		

*Notes*: Symbols in this table denote the status of causal conditions within each sufficient configuration, interpreted as follows: ⬤ (Large solid circle): Indicates the presence of a core condition. ⨂ (Large circle with cross): Indicates the absence of a core condition. ● (Small solid circle): Indicates the presence of a peripheral condition. ⊗ (Small circle with cross): Indicates the absence of a peripheral condition. Blank space: Indicates a “don’t care” condition (the condition’s presence or absence does not impact the sufficiency of this specific configuration).

In contrast, for non-high-severity outcomes, configurations N-CD1, N-CI1, and N-CI3 identify the absence of feedback errors as core. N-CI2 provides an alternate path to avoid injuries by requiring the absence of controller failures alongside the presence of controlled process errors. For avoiding high economic loss, N-CE1 specifies the absence of control activity errors, controller failures, and controlled process errors, while N-CE2 and N-CE3 again emphasize the absence of feedback errors as central.

Interpreting the parameters of fit (i.e., consistency and coverage) clarifies each configuration’s reliability and empirical relevance. High consistency (≥0.80) indicates a robust pathway toward an outcome (e.g., CE1 and CE2 for high economic loss), whereas raw coverage highlights how frequently each configuration appears among outcome cases, and unique coverage shows the proportion of cases explained exclusively by that configuration. The overall solution consistency and solution coverage further demonstrate how well the combined configurations explain each outcome.

A central finding is that feedback errors consistently appear in high-severity pathways, underscoring their critical role in accident escalation. Effective feedback mechanisms are crucial to reducing injuries and minimizing economic loss, in tandem with reducing control activity errors, controller failures, and controlled process errors. At lower severity, none of these factors appear salient, but they gain prominence as severity increases.

**Fig 6:**
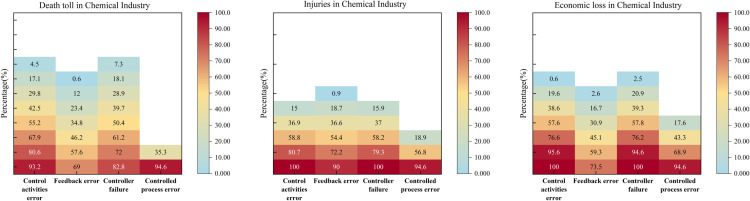
Performance of the NCA bottleneck level in the chemical industry.

Fig 6’s NCA analysis confirms that feedback errors exert disproportionate influence at even modest levels. For instance, a 40% death rate may be triggered by as little as a 0.6% increase in feedback errors, whereas control activity errors and controller failures must reach higher thresholds (17.1% and 18.1%, respectively).

Real-world incidents, such as the “3·21” major explosion at Jiangsu Tianjiayi Chemical Co., Ltd., illustrate this dynamic. Long-term illegal hazardous waste storage and flawed evaluation reports exacerbated functional failures. Communication and feedback deficiencies across administrative, design, and governmental bodies further weakened safety constraints. Strengthening these communication channels is thus essential to enhance safety performance and prevent severe accidents in the chemical industry.

### 3.2. Construction industry

Table 4 summarizes the QCA findings for the construction industry. Configuration BD1 (high death toll) highlights the joint presence of control activity errors, feedback errors, and controller failures as core conditions. For high injuries, three configurations emerge: (1) BI1 requires feedback errors and controller failures; (2) BI2 requires control activity errors and controller failures; and (3) BI3 involves control activity errors but the absence of controller failures. Meanwhile, BE1 (high economic loss) underscores the presence of control activity errors and controller failures.

**Table 4:** The construction industry has a high severity of accident development and non-high severity of accident development conditions.

Configuration	High severity of accident development					Non-high severity of accident development							
Death toll	Injuries			Economic loss	~Death toll		~Injuries			~Economic loss		
BD1	BI1	BI2	BI3	BE1	N-BD1	N-BD2	N-BI1	N-BI2	N-BI3	N-BE1	N-BE2	N-BE3
Control activities error	⬤	⊗		⬤		⨂			⊗	⊗		⊗	⊗
Feedback error	⬤		●	⊗	⬤		⨂	⨂		⨂	⨂		⨂
Controller failure	⬤	⬤	⬤	⊗	⬤		⨂	⨂	⨂		⨂	⨂	
Controlled process error	●	⊗	●	●	●	●	●	●	●	⊗	●	●	●
Consistency	0.864	0.830	0.722	0.773	0.946	0.990	0.992	0.894	0.884	0.897	0.994	0.996	0.996
Raw coverage	0.776	0.513	0.672	0.328	0.852	0.654	0.592	0.540	0.546	0.540	0.615	0.631	0.621
Unique coverage	0.777	0.098	0.316	0.017	0.852	0.066	0.004	0.003	0.009	0.008	0.003	0.019	0.009
Solution consistency	0.864	0.758			0.946	0.993		0.885			0.994		
Solution coverage	0.777	0.849			0.852	0.659		0.558			0.644		

High consistency values (e.g., BD1 = 0.864, BE1 = 0.946) indicate that these configurations reliably lead to severe outcomes. Raw coverage (BD1 = 0.776, BE1 = 0.852) suggests these pathways explain most observed cases. Unique coverage shows BD1 (0.777) and BE1 (0.852) have distinct explanatory power for deaths and economic loss, whereas the injury pathways share more overlap (e.g., BI1 = 0.098, BI3 = 0.017). Overall solution consistency (e.g., 0.864 for deaths) confirms the collective sufficiency of identified pathways, while solution coverage (0.777 for deaths, 0.849 for injuries, 0.852 for economic loss) demonstrates their high explanatory power.

Two main points emerge. First, controller failures combined with either control activity errors or feedback errors greatly increase accident severity. Second, configurations with either control activity errors or both feedback errors and controller failures capture a large share of severe accidents. Thus, focusing on minimizing control activity errors alone or jointly reducing feedback errors and controller failures can be an efficient strategy for practitioners.

Studies show that controller and feedback errors often co-occur in construction. Many firms lack systematic feedback on regulatory compliance, and legal oversight mechanisms are limited, making it easy for repeated mistakes to occur [48]. Addressing these failures together thus targets both a major accident cause and a common weakness.

**Fig 7:**
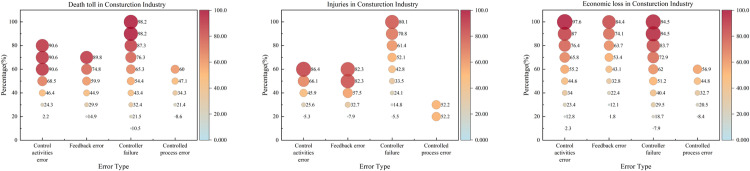
NCA bottleneck level performance in the construction industry.

Fig 7 reveals that controller failures frequently arise early in minor injury accidents, reflecting organizational shortcomings more than external factors. Research indicates that “delay in hazard removal” and “insufficient safety inspections” are critical issues [49]. Leadership plays a key role in mitigating these failures by setting standards, identifying system errors, and closely monitoring risks [50].

The 2016 Fengcheng power plant collapse in Jiangxi caused 73 deaths, 2 injuries, and a direct economic loss of 101.972 million yuan [49]. Under tight deadlines, workers prematurely removed formwork before the concrete reached adequate strength, triggering a fast chain collapse. The accident was attributed to “non-compliance with technical specifications” and “disobedience of regulations,” underscoring the importance of adhering to safety guidelines and ensuring proper oversight.

In contrast to the chemical industry, controlled process errors appear at moderate severity levels in construction, although they do not always emerge as core conditions in QCA. Nonetheless, these errors act as significant peripheral factors, influencing accident severity alongside control activity and feedback errors.

### 3.3. Transportation industry

Table 5 demonstrates that in high fatality cases (TD1), only the presence of control activity errors is a core condition. For high injuries, both TI1 and TI2 require control activity errors and feedback errors, while TI3 combines control activity errors with the absence of controller failures. For high economic loss, TE1 and TE3 highlight controller failures and the absence of controlled process errors as core, whereas TE2 and TE4 emphasize control activity errors and feedback errors.

**Table 5:** The transportation industry has a high severity of accident development and a non-high severity of accident development.

Configuration	High severity of accident development										Non-high severity of accident development						
Death toll			Injuries			Economic loss				~Death toll		~Injuries		~Economic loss		
TD1	TD2	TD3	TI1	TI2	TI3	TE1	TE2	TE3	TE4	N-TD1	N-TD2	N-TI1	N-TI2	N-TE1	N-TE2	N-TE3
Control activities error	⬤	⬤	⬤	⬤	⬤	⬤	⬤	⬤	⬤	⬤	⨂	⨂	⨂	●	⨂	●	⨂
Feedback error		⊗	⬤	⬤	●	⊗	⊗	⬤		⬤	⊗	●	⨂	⨂	⊗	⨂	●
Controller failure	●			●		⨂			●	●	⊗	●	⨂	⬤	⊗	●	●
Controlled process error		⊗	●		●	⊗	⨂	●	⊗			●				⬤	●
Consistency	0.887	0.905	0.964	0.887	0.938	0.823	0.832	0.915	0.934	0.927	0.946	0.938	0.929	0.870	0.941	0.918	0.872
Raw coverage	0.741	0.447	0.504	0.664	0.524	0.360	0.450	0.523	0.609	0.710	0.660	0.268	0614	0.342	0.611	0.220	0.232
Unique coverage	0.132	0.054	0.049	0.104	0.052	0.055	0.035	0.054	0.021	0.023	0.482	0.090	0.428	0.156	0.457	0.091	0.061
Solution consistency	0.880			0.860			0.887				0.935		0.937		0.927		
Solution coverage	0.850			0.777			0.867				0.751		0.771		0.781		

In non-high-severity scenarios, avoiding fatalities (N-TD1) necessitates the absence of control activity errors. Avoiding injuries (N-TI1) requires no control activity errors, no feedback errors, and no controller failures. For avoiding high economic loss, N-TE1 and N-TE3 again stress the absence of control activity errors, while N-TE2 focuses on the absence of feedback errors alongside controlled process errors.

Most configurations show high consistency (≥0.82), confirming their reliability in predicting outcomes. Variations in raw coverage reveal differences in how frequently each pathway occurs, while unique coverage underscores each path’s distinct explanatory power. Across outcomes, overall solution consistency and coverage remain high (0.860–0.937 and 0.751–0.867, respectively), indicating these pathways collectively explain most severe and non-severe accidents in the transportation dataset.

Control activity errors strongly drive severe road accidents, with feedback errors playing a notable role, especially in injuries and economic losses. While reducing control activity errors is paramount, feedback errors and controller failures also demand attention. Compared to the chemical and construction sectors, controller failures and controlled process errors appear less frequently, partly because individual driver behavior, not complex organizational factors, often precipitates road incidents.

**Fig 8:**
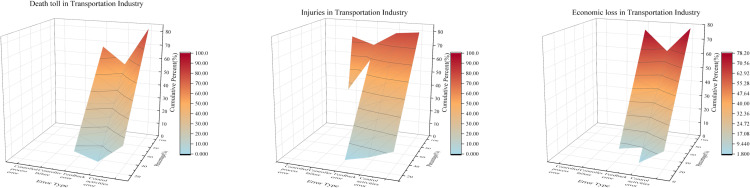
NCA bottleneck level performance in the transportation industry.

Fig 8 confirms that control activity errors surface early in accident progression, aligning with their QCA status as a core condition. Controlled process errors show minimal influence on fatalities and economic loss, further underscoring the pivotal role of control activities in preventing road traffic accidents.

Globally, road safety responsibilities—vehicle checks, infrastructure design, rule enforcement—are spread across multiple agencies [51,52]. Weak coordination or oversight can leave errors unchecked, increasing accident risks. In a 2016 crash on the Yifeng Expressway, driver fatigue and illicit company operations (e.g., lacking safety inspections) amplified risks, and regulatory gaps allowed violations to persist. Unlike aviation and rail, road transport lacks similarly stringent controls. Strengthening regulations, monitoring, and supervisory mechanisms is thus essential, potentially through innovative control strategies that improve safety without overly burdening road users.

### 3.4. Coal mining industry

Table 6 highlights core conditions driving severe accidents in coal mining. Fatalities consistently involve controller failures and controlled process errors. Injuries emerge from multiple pathways, reflecting their multifaceted causes. In high economic loss scenarios, ME1 and ME3 pinpoint control activity errors as core, while ME2 uniquely focuses on the absence of feedback errors, controller failures, and controlled process errors. Configuration M34 combines control activity errors, controller failures, and controlled process errors.

**Table 6:** The coal mining industry has a high severity of accident development and a non-high severity of accident development condition.

Configuration	High severity of accident development							Non-high severity of accident development								
Death toll		Injuries			Economic loss		~Death toll			~Injuries				~Economic loss	
MD1	MD2	MI1	MI2	MI3	ME1	ME2	N-MD1	N-MD2	N-MD3	N-MI1	N-MI2	M-MI3	N-MI4	N-ME1	N-ME2
Control activities error		●	⬤	⬤		⬤	⬤		⊗	⨂		⨂	⨂		⨂	⨂
Feedback error	⊗		⬤	●	⨂	●		●		⬤	●		●	⨂		●
Controller failure	⬤	⬤	⬤		⬤		⬤	⨂	⨂		⨂	⨂		●	⨂	⨂
Controlled process error	⬤	⬤	⬤	⊗	⬤	●	⬤		⨂	⨂		⨂	⨂	●	⊗	
Consistency	0.887	0.969	0.833	0.898	0.874	0.959	0.909	0.975	0.968	0.900	0.927	0.884	0.918	0.851	0.927	0.914
Raw coverage	0.457	0.577	0.428	0.319	0.440	0.431	0.528	0.560	0.736	0.549	0.442	0.558	0.465	0.328	0.543	0.710
Unique coverage	0.050	0.170	0.428	0.091	0.043	0	0.030	0.041	0.217	0.030	0.030	0.117	0.042	0.143	0.040	0.207
Solution consistency	0.915		0.833	0.849				0.920			0.889				0.918	
Solution coverage	0.628		0.428	0.725				0.808			0.793				0.750	

Paths for avoiding fatalities show the absence of controller failures (N-MD1) or the absence of both controller failures and controlled process errors (N-MD2). N-MD3 integrates the absence of control activities errors, the presence of feedback errors, and the absence of controlled process errors. Similar variety appears in avoiding injuries (N-MI1 through N-MI4) and avoiding high economic loss, where the absence of control activities errors and controller failures repeatedly emerges as core.

Most configurations exhibit high consistency (>0.80), indicating they reliably predict outcomes. Raw coverage varies, while unique coverage clarifies each path’s distinct explanatory reach. Overall solution consistency (0.833–0.920) and solution coverage (0.628–0.808) confirm robust explanatory power.

**Fig 9:**
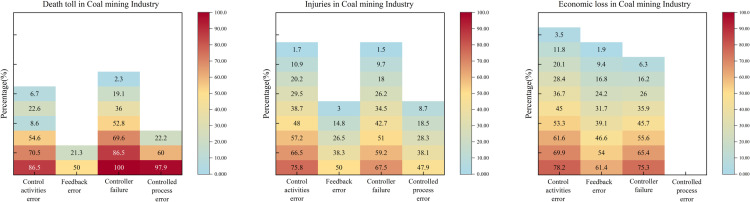
NCA bottleneck level performance in the coal mining industry.

Fig 9 indicates control activity errors and controller failures significantly influence coal mining accidents, even at lower severity levels. Just 5.3% of control activity errors can lead to a 100% direct economic loss, highlighting the need to prioritize these two factors. Compared to other industries, the coal sector remains highly prone to mid- and low-level accidents with elevated casualty risks.

A severe 2016 gas explosion at Baoma Mining Co. (Inner Mongolia) caused 32 fatalities and 20 injuries [53]. Falsified documents concealed illegal mining, underscoring systemic lapses in safety supervision and inspection. Common risk contributors include unscientific supervision plans, shortfalls in technical personnel, falsified monitoring data, and regulatory negligence [54]. Strengthening control activities and enhancing controller oversight—through comprehensive regulations, consistent safety checks, and robust monitoring—remain critical to reducing accidents and securing safer coal mining operations [55,56].

### 3.5. Firefighting industry

Table 7 shows that for high-severity outcomes—high fatalities (FD1), high injuries (FI1), and high economic losses (FE1)—the combined presence of control activity errors and controller failures consistently emerges as core. Conversely, in non-high-severity cases (~low severity), avoiding fatalities (N-FD1) or injuries (N-FI1) requires the absence of both control activity errors and controller failures, while avoiding high economic loss (N-FE1) entails the absence of control activity errors, controller failures, and controlled process errors.

**Table 7:** The firefighting industry has a high severity of accident development and non-high severity of accident development conditions.

Configuration	High severity of accident development			Non-High severity of accident development			
Death toll	Injuries	Economic loss	~Death toll	~Injuries	~Economic loss	
FD1	FI1	FE1	N-FD1	N-FI1	N-FE1	N-FE2
Control activities error	⬤	⬤	⬤	⨂	⨂	⨂	⬤
Feedback error							
Controller failure	⬤	⬤	⬤	⨂	⨂	⨂	⬤
Controlled process error	●	●	●	●	●	⬤	⬤
Consistency	0.886	0.838	0.662	0.991	0.966	0.912	0.769
Raw coverage	0.877	0.798	0.668	0.619	0.620	0.563	0.506
Unique coverage	0.877	0.798	0.668	0.619	0.620	0.212	0.155
Solution consistency	0.886	0.838	0.662	0.991	0.966	0.781	
Solution coverage	0.877	0.798	0.668	0.619	0.620	0.718	

Key high-severity pathways (FD1 = 0.886, FI1 = 0.838) demonstrate high consistency, reliably predicting severe outcomes. Raw coverage values (e.g., FD1 = 0.877, FI1 = 0.798) indicate these pathways explain most cases of severe accidents. FE1 has slightly lower consistency (0.662) but still a high raw coverage of 0.668. Non-high-severity paths—N-FD1 = 0.991 and N-FI1 = 0.966—also reveal strong consistency, underlining that avoiding control activity errors and controller failures is highly effective for reducing fatalities and injuries. Overall solution coverage (e.g., 0.877 for high fatalities) underscores the explanatory power of these single dominant pathways.

The presence or absence of control activity errors and controller failures emerges as the central differentiator in most firefighting accidents. However, research often focuses only on immediate fire management, overlooking how building structures, environmental factors, and organizational oversights (e.g., inadequate maintenance of fire systems, weak regulatory enforcement) drive accidents [57]. Construction site fires also highlight neglected fire-safety systems, often tied to internal controller failures [58].

A major fire accident in Zhecheng County, Henan Province, revealed years of lax regulatory oversight, where neglect by multiple agencies and violations at the fire site went undetected. The blaze ignited from a mosquito coil in a poorly monitored environment, illustrating how weak daily supervision and noncompliance with safety standards can lead to severe consequences.

**Fig 10:**
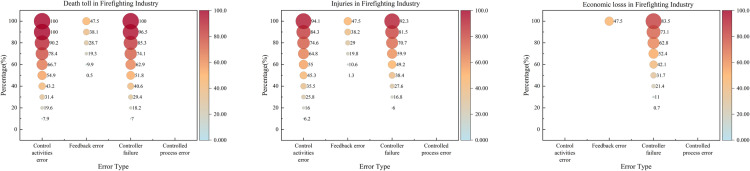
NCA bottleneck level performance in a firefighting industry.

Fig 10 indicates that even a 73.6% level of controller failures alone can trigger a 90% direct economic loss, underscoring controller failures as a primary driver of financial impacts. For casualties, control activity errors and controller failures also appear early, consistent with the QCA findings.

Reducing casualties in firefighting accidents requires correcting control activity errors through stronger supervision and inspections. Meanwhile, preventing economic losses necessitates addressing underlying controller weaknesses. Achieving higher fire safety standards demands systemic reforms, including robust oversight, heightened investment in firefighting facilities, and internal controller enhancements to mitigate both economic and human losses.

## 4. Comparative analysis

### 4.1. Comparative analysis of core conditions

**Fig 11:**
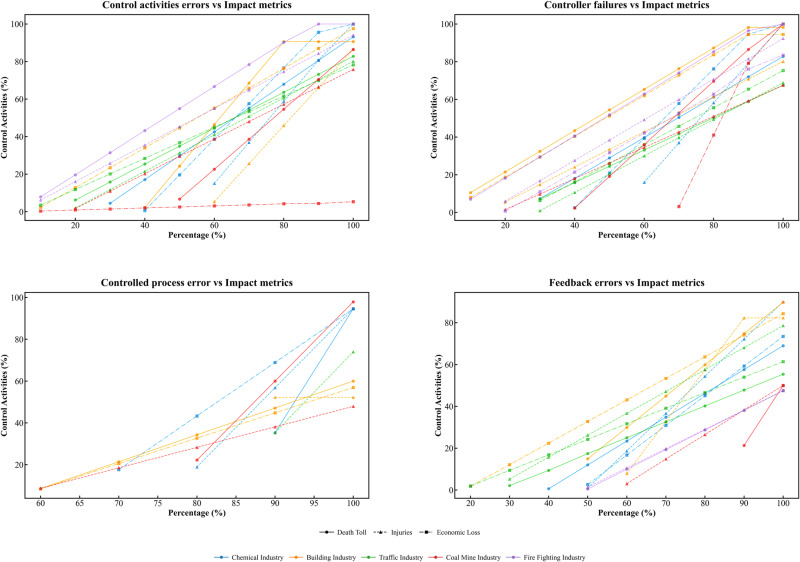
The impact of core conditions varies across different industries.

Comparative analysis of control activities errors, feedback errors, controller faults, and controlled process errors across five industries, as shown in Fig 11, reveals the varying manifestations and impact levels of these core conditions in different industries. We identified distinct patterns in their occurrences as well as some common characteristics across these sectors.

#### 4.1.1. Control activities error.

In the chemical industry, control behavior errors appear relatively late, particularly in terms of economic losses, only becoming evident when the accident severity reaches 40%. This may be due to the complexity of the chemical industry and the delayed response at the regulatory level, leading to the late identification and handling of control behavior issues. Therefore, it is necessary to strengthen early identification and intervention measures, such as enhancing supervision and improving accident prevention systems.

In contrast, control behavior errors manifest in minor accidents in the construction, coal mining, and fire protection industries, particularly in the fire protection industry, where control behavior errors appear when accident severity is as low as 10%. This suggests that the control mechanisms in these industries may be inadequate, resulting in the failure to timely correct potential errors [59]. It is recommended to implement stricter control behavior supervision and review mechanisms and to strengthen the application of feedback from accident and incident analysis.

The construction and fire protection industries show a high degree of similarity in the impact of control behavior errors, possibly due to the direct correlation between control activities and controlled processes in these two industries. For example, construction operations and fire rescue operations both rely heavily on stringent process control. Enhancing the effectiveness of controllers and reducing control behavior errors are particularly critical.

The early manifestation of control behavior errors in the fire protection industry is particularly pronounced, highlighting the special needs for control behavior management in this sector. Fire departments need to strengthen real-time monitoring and dynamically adjust control strategies to ensure rapid and effective responses in emergency situations.

#### 4.1.2. Feedback errors.

In the chemical industry, feedback errors typically do not manifest until the accident severity reaches 40%. This delay may be attributed to the complex processes and multi-layered monitoring systems within the chemical industry, causing a delayed response in the feedback mechanisms. However, feedback errors significantly impact economic losses, with this impact becoming more pronounced at higher accident severities. This is likely due to the high chain reaction and aftermath costs associated with chemical accidents. The chemical industry should enhance real-time monitoring systems and early warning mechanisms to improve the timeliness and accuracy of feedback [60].

In the construction and transportation industries, feedback errors affect injuries and economic losses at an earlier stage. This indicates that the feedback systems in these industries are closely related to daily operations, and erroneous feedback can quickly impact operational safety and costs. In the construction industry, in particular, due to the time sensitivity of projects and the need for cost control, timely and accurate feedback is crucial. These industries need to adopt more efficient data collection and analysis tools to improve feedback and monitoring systems throughout the entire lifecycle.

In the transportation industry, the impact of feedback errors on accident severity is relatively low. This may be because the direct causes of transportation accidents are more related to the immediate decisions of operators rather than systemic feedback failures. It is recommended that the transportation industry focus on improving real-time monitoring and operator training to reduce human errors, rather than relying solely on post-incident feedback.

#### 4.1.3. Controller failures.

In the coal mining and construction industries, the impact of controller failures on accidents manifests very early, reflecting the importance of control systems and their direct influence on safety in these sectors. Since these industries rely on complex machinery and equipment operations, controller failures can quickly lead to severe safety incidents, such as gas explosions in coal mines or structural collapses in construction. These industries should enhance regular maintenance and upgrades of control systems to ensure the reliability and responsiveness of controllers [61].

In the chemical industry, the impact of controller failures on economic losses appears later. This delay might be due to the fact that the consequences of chemical industry accidents often take time to fully manifest; for instance, leaked chemicals might take time to cause equipment damage or environmental pollution. The chemical industry should emphasize long-term monitoring and preventive measures, strengthening post-accident impact assessments and response strategies.

In the fire protection industry, the impact of controller failures on economic losses becomes evident at lower stages of accident severity. This is likely because fire protection tasks typically involve emergency responses, and controller failures can lead to delays or failures in rescue operations, rapidly escalating economic losses and personal injuries. It is recommended that the fire protection industry adopt high-standard controller designs and redundancy systems to ensure efficient operations during emergencies.

#### 4.1.4. Controlled process error.

In the chemical and coal mining industries, controlled process errors typically do not manifest until the accident severity is high. This reflects the challenges in managing and supervising complex and hazardous operations in these sectors. The operations in these industries are intricate and associated with high risks; the consequences of errors may take time to become apparent, but once they do, the impacts are often catastrophic. The chemical and coal mining industries should take measures to strengthen the supervision and auditing of controlled processes to ensure the correct and effective execution of all control commands.

The chemical and coal mining industries exhibit a high degree of similarity in the impact of controlled process errors, particularly in terms of economic losses. Both industries rely on precise and reliable operational processes, and errors in controlled processes directly affect production efficiency and costs. This necessitates the implementation of meticulous quality control and risk assessment throughout the entire operation process, as well as the establishment of robust feedback and corrective action systems.

In contrast, in the construction industry, the impact of controlled process errors on economic losses is relatively marginal. This may be because construction projects typically have well-defined stages and checkpoints, allowing errors in controlled processes to be corrected in a timely manner without affecting the overall project. The construction industry should continue to promote best practices in project management, such as regular reviews and phase-based quality inspections, to ensure that all control activities are properly executed.

In the transportation industry, the impact of controlled process errors is almost negligible. This might indicate that accidents in this industry are more driven by immediate decision-making errors or external environmental factors rather than long-term controlled process management issues. The transportation industry should enhance real-time monitoring and training for operators to improve their response capabilities in emergencies, as well as enhance the technical support for vehicles and traffic management systems.

### 4.2. Key conditions in accident development stages

When analyzing accident development processes in the chemical, construction, transportation, coal mining, and fire protection industries, we observed that the manifestation and impact of key conditions vary according to accident severity. By examining these key conditions, we can more precisely identify each industry’s management priorities and strategies at different severity levels.

#### 4.2.1. Low-severity accidents.

In low-severity accidents, control activities errors and controller failures are the key factors leading to accidents. Control activities are conveyed from higher-level organizations to lower-level ones, and a failure or improper execution at any level can trigger initial accidents. Although the impacts at this stage tend to be relatively minor, they can escalate into more serious situations if not corrected promptly.

During this stage, organizational feedback mechanisms play a vital preventive role. Through audits, reporting, and analyses of accidents and near-miss events, controllers can adjust control measures in a timely manner to avert further escalation [62]. A well-functioning feedback system can detect potential risks early on and take measures to prevent the accident from worsening.

Consistent with earlier human factors analyses [63], the current study similarly identifies the importance of promptly detecting individual control lapses and organizational oversight weaknesses in preventing the progression of early-stage accidents. Prior research has also stressed that rapid intervention to address such initial lapses can significantly reduce the likelihood of an accident deteriorating [64]. Furthermore, although some studies have shown that constrained analytical scope or incomplete data can lead to underestimation of higher-level influences [65], our multi-industry findings suggest that effectively implementing feedback and audit mechanisms at this early stage can reveal and address overlooked higher-level organizational or regulatory breakdowns. This aligns with broader audit-based practices that emphasize proactive risk identification [66,67].

#### 4.2.2. High-severity accidents.

In high-severity accidents, the stability of the controlled process and the continuous management capability of controllers are critical. Because of cumulative effects [68], errors in the controlled process can lead to severe outcomes such as major safety incidents or environmental pollution. Failures in long-term control activities or malfunctions in actuators are common in this context, indicating that controllers are unable to manage or adjust the controlled process effectively.

Feedback mechanisms at this stage should focus on in-depth analysis of the root causes of accidents and systemic improvements. Systematic accident analyses and audits can pinpoint systemic deficiencies within the control process and help prevent future occurrences. In addition, feedback mechanisms should continuously monitor small, persistent issues to prevent them from developing into major safety hazards.

Previous studies have shown that when early, minor errors remain unaddressed for an extended period, they tend to accumulate and lead to severe consequences when a major accident occurs [69]. Our multi-industry empirical evidence reinforces this conclusion, aligning with other work that has highlighted the role of higher-level management lapses in amplifying accident severity [70]. Moreover, unlike earlier studies focusing predominantly on a single sector [71], this research—examining chemical, construction, transportation, coal mining, and fire protection industries—reveals that the absence of sustained top-level oversight and systematic feedback amplifies the cumulative impact of minor issues. Therefore, regardless of whether an incident initially centers on human error or technical failure, if an organization does not effectively utilize feedback and take timely preventive actions at higher-level control layers, severe or catastrophic outcomes may ultimately ensue [72].

### 4.3. Comparative analysis with statistical prioritization methods

To further contextualize this study’s configurational findings and address the reviewer’s request for statistical factor prioritization, supplementary analyses (Spearman correlation, exploratory multiple regression) were performed across the five industries (N = 16 each; Table 8 Combined). Spearman correlations generally highlighted Control Activities Error and Controller Failure as strongly associated with Deaths and Economic Loss in most sectors, while Feedback Error showed strong correlations across outcomes, particularly in Chemical and Transportation. Controlled Process Error typically exhibited weaker associations.

**Table 8:** Combined: summary of statistical analysis results for control factors and accident severity across five industries.

Industry	Analysis method	Outcome	Control activities error	Feedback error	Controller failure	Controlled process error	Model adj. R²
Chemical [a]	Spearman Corr (ρ)	DT	0.734***	0.874***	0.602**	0.552**	–
		IN	0.301	0.628**	0.276	0.307	–
		log_EL	0.611**	0.738***	0.547**	0.645***	–
	Regression (Std. β)	DT	1.419***	0.135	−0.805†	−0.196	0.517
		IN	1.522***	0.085	−1.016**	−0.165	0.519
		log_EL	0.986**	0.230	−0.664	0.094	0.332
Construction[b]	Spearman Corr (ρ)	DT	0.824***	0.655**	0.889***	0.792***	–
		IN	0.310	0.365	0.501*	0.269	–
		log_EL	0.950***	0.812***	0.875***	0.818***	–
	Regression (Std. β)	DT	1.147***	−0.252†	0.083	−0.150	0.894
		IN	0.190	0.799*	−0.512	0.252	0.390
		log_EL	0.691	0.542	−0.412	−0.088	0.340
Transportation[c]	Spearman Corr (ρ)	DT	0.883***	0.708**	0.707**	−0.079	–
		IN	0.465†	0.786***	0.308	0.165	–
		log_EL	0.677**	0.713**	0.565*	−0.091	–
	Regression (Std. β)	DT	0.907***	0.092	−0.165	−0.221	0.725
		IN	0.278	0.764***	−0.250	0.056	0.546
		log_EL	0.573†	0.305	−0.190	−0.052	0.237
Coal Mining [d]	Spearman Corr (ρ)	DT	0.714**	−0.144	0.856***	0.535*	–
		IN	0.502*	0.094	0.363	0.226	–
		log_EL	0.626**	−0.092	0.662**	0.367	–
	Regression (Std. β)	DT	0.755*	−0.095	0.555**	−0.296	0.808
		IN	0.149	0.175	0.139	0.065	−0.160
		log_EL	0.412	−0.134	0.508†	−0.272	0.194
Firefighting [e]	Spearman Corr (ρ)	DT	0.960***	0.777***	0.908***	0.549*	–
		IN	0.911***	0.726**	0.793***	0.397	–
		log_EL	0.300	0.287	0.369	−0.047	–
	Regression (Std. β)	DT	0.393	−0.109	0.684*	−0.270*	0.884
		IN	0.410	0.021	0.545	−0.335*	0.718
		log_EL	0.154	−0.084	0.253	−0.211	−0.185

*Notes*: N = 16 for each industry. Correlation analysis uses Spearman’s rho (ρ). Regression analysis uses Standardized Beta coefficients (Std. β); *** p < 0.001, ** p < 0.01, * p < 0.05, † p < 0.1. log_EL is the log-transformed value of direct economic loss.

[a] Chemical: Regression models exhibit moderate multicollinearity (Controller Failure VIF = 5.6, Control Activities Error VIF = 4.3, Controlled Process Error VIF = 4.2).

[b] Construction: Regression models exhibit significant multicollinearity (Control Activities Error VIF = 7.5, Controller Failure VIF = 8.6).

[c] Transportation: Multicollinearity is low (All VIFs < 1.7).

[d] Coal Mining: Regression models exhibit significant multicollinearity (Control Activities Error VIF = 7.0, Controlled Process Error VIF = 7.1). Injury (IN) regression model is invalid (Adj. R² < 0) due to highly skewed data.

[e] Firefighting: Regression models exhibit severe multicollinearity (Control Activities Error VIF = 12.8, Controller Failure VIF = 10.8). Log Economic Loss (log_EL) regression model is invalid (Adj. R² < 0). Injury (IN) regression results potentially influenced by outlier (Case 15, IN=76).

However, interpreting the independent contributions via regression was significantly hampered by small sample sizes and, in several industries (notably Construction, Coal Mining, Firefighting), severe multicollinearity, rendering some models unstable or invalid. Where interpretable, regression tentatively pointed towards Control Activities Error as a key predictor for Deaths (e.g., Chemical, Construction, Transportation) and Feedback Error for Injuries (e.g., Construction, Transportation), though these findings must be viewed with extreme caution.

Comparing these statistical results with the primary fsQCA/NCA findings reveals important complementarities and highlights the unique value of the configurational approach adopted in this research. There is convergence in identifying Control Activities Error and Controller Failure as critical risk factors, supported by both strong correlations and their frequent appearance as core conditions in fsQCA pathways leading to severe outcomes. Similarly, Feedback Error’s importance, especially in Chemical and Transportation, was echoed by both methods. However, the comparison also underscores the limitations of relying solely on statistical methods under these conditions. Regression struggled to disentangle the effects of highly collinear factors (e.g., Control Activities Error and Controller Failure in Firefighting), whereas fsQCA naturally assesses their combined effect as a causal configuration. Furthermore, fsQCA revealed nuanced roles for factors like Feedback Error (important in preventing accidents in some contexts) and Controlled Process Error (critical mainly in specific configurations associated with higher severity, particularly in Chemical and Coal Mining) that were obscured in the average-effect statistical models.

The configurational perspective offered by fsQCA/NCA proves particularly adept at handling the causal complexity inherent in accident analysis. It explicitly accounts for equifinality (multiple pathways to the same outcome), identifies the significance of condition absence (asymmetry), and, through NCA, provides insights into necessity thresholds – aspects often challenging for standard linear models, especially with limited data and interacting variables. While statistical correlations provide a useful initial indication of factor importance, they do not fully capture the combinatorial and context-dependent nature of systemic failures.

In conclusion, this comparative analysis demonstrates that while supplementary statistical tests offer some corroborating evidence for the key risk factors identified, the STAMP-based fsQCA/NCA methodology provides a more robust, nuanced, and theoretically grounded understanding of how combinations of control failures lead to severe accidents across the studied industries. By embracing causal complexity, this approach offers valuable insights beyond traditional statistical prioritization, strengthening the foundation for developing effective, system-oriented safety interventions.

## 5. Conclusion

This study integrated the Systems-Theoretic Accident Model and Processes (STAMP) framework with Qualitative Comparative Analysis (QCA) and Necessary Condition Analysis (NCA) to examine major accident reports from five high-risk Chinese industries. Focusing on control activities errors, feedback errors, controller failures, and controlled process errors, we identified complex causal configurations influencing accident severity.

Our analysis revealed industry-specific pathways and common themes, such as the early influence of control activity and controller failures, the significant role of feedback errors (especially in the chemical sector), and the association of controlled process errors with high-severity outcomes. These findings highlight the staged nature of accident development, demanding tailored systemic interventions.

The study’s main contribution lies in applying a configurational, systems-theoretic lens (STAMP + fsQCA/NCA) to understand how combinations of control failures drive accident severity. A comparative analysis against traditional statistical methods (Section 4.3) confirmed the value of this approach, demonstrating fsQCA/NCA’s capacity to provide robust insights into combinatorial effects and causal pathways, particularly given the data limitations (small N, multicollinearity) common in accident research that challenged statistical models.

Limitations remain. Methodologically, QCA/NCA imply causality and offer a static, configurational view, differing from net-effect statistics or dynamic models like System Dynamics (SD). Results depend on calibration choices, and the scope was confined to four broad factors and specific Chinese contexts/data. Acknowledging alternatives, traditional statistics struggle with equifinality, while Fuzzy logic [73]and MCDM methods [74–76] offer other strengths. Our STAMP+QCA/NCA approach was chosen, and demonstrated its utility, for uncovering causal recipes within a systems framework [76].

Future research should pursue methodological integration (e.g., QCA with process tracing or DEMATEL), compare findings using different systemic or computational models [75,77], employ mixed-methods designs for richer data, broaden the scope of conditions, and test generalizability. Such efforts will build upon this study’s configurational insights into the systemic control failures underlying severe accidents.

## Supporting information

S1 Appendix Template of the official accident investigation report. (DOCX)

S2 Dataset Eighty accident cases from 2010–2022. (DOCX)

## References

[1] Dekker S, Pitzer C. Examining the asymptote in safety progress: a literature review. Int J Occup Saf Ergon. 2016;22(1):57–65. doi: 10.1080/10803548.2015.1112104 26652223

[2] Zhou J-L, Tu R-F, Xiao H. Large-scale group decision-making to facilitate inter-rater reliability of human-factors analysis for the railway system. Reliabil Eng Syst Safe. 2022;228:108806. doi: 10.1016/j.ress.2022.108806

[3] La Fata CM, Adelfio L, Micale R, La Scalia G. Human error contribution to accidents in the manufacturing sector: a structured approach to evaluate the interdependence among performance shaping factors. Safe Sci. 2023;161:106067. doi: 10.1016/j.ssci.2023.106067

[4] Salmon PM, Hulme A, Walker GH, Waterson P, Stanton NA. Towards a unified model of accident causation: refining and validating the systems thinking safety tenets. Ergonomics. 2023;66(5):644–57. doi: 10.1080/00140139.2022.2107709 35902801

[5] Hulme A, Stanton NA, Walker GH, Waterson P, Salmon PM. Complexity theory in accident causation: using AcciMap to identify the systems thinking tenets in 11 catastrophes. Ergonomics. 2021;64(7):821–38. doi: 10.1080/00140139.2020.1869321 33357083

[6] Li J, Wang H. Modeling and analyzing multiteam coordination task safety risks in socio-technical systems based on FRAM and multiplex network: application in the construction industry. Reliabil Eng Syst Safe. 2023;229:108836. doi: 10.1016/j.ress.2022.108836

[7] Ma Z, Chen Z-S. Mining construction accident reports via unsupervised NLP and Accimap for systemic risk analysis. Automat Construct. 2024;161:105343. doi: 10.1016/j.autcon.2024.105343

[8] Gholamizadeh K, Alauddin M, Aliabadi MM, Soltanzade A, Mohammadfam I. Comprehensive failure analysis in Tehran refinery fire accident: application of Accimap methodology and quantitative domino effect analysis. Fire Technol. 2022;59(2):453–72. doi: 10.1007/s10694-022-01348-6

[9] Shappell SA, Wiegmann DA. Applying reason: the human factors analysis and classification system (HFACS). Human factors and aerospace safety. 2001 [cited 16 Oct 2023]. Available: https://psycnet.apa.org/record/2003-02913-002.

[10] Yousefi A, Rodriguez Hernandez M, Lopez Peña V. Systemic accident analysis models: a comparison study between AcciMap, FRAM, and STAMP. Process Safe Progr. 2018;38(2). doi: 10.1002/prs.12002

[11] Hulme A, Stanton NA, Walker GH, Waterson P, Salmon PM. What do applications of systems thinking accident analysis methods tell us about accident causation? A systematic review of applications between 1990 and 2018. Safe Sci. 2019;117:164–83. doi: 10.1016/j.ssci.2019.04.016

[12] Wu Y, Zhang S, Zhang X, Lu Y, Xiong Z. Analysis on coupling dynamic effect of human errors in aviation safety. Accid Anal Prev. 2023;192:107277. doi: 10.1016/j.aap.2023.107277 37690283

[13] Ma L, Ma X, Wang T, Zhao Y, Lan H. A data-driven approach to determine the distinct contribution of human factors to different types of maritime accidents. Ocean Eng. 2024;295:116874. doi: 10.1016/j.oceaneng.2024.116874

[14] Junjia Y, Alias AH, Haron NA, Abu Bakar N. Identification and analysis of hoisting safety risk factors for IBS construction based on the AcciMap and cases study. Heliyon. 2024;10(1):e23587. doi: 10.1016/j.heliyon.2023.e23587 PMC10772131 38192814

[15] Zheng Q, Liu X, Wang W. A consensus model-based risk matrix for human error factors risk analysis in medical devices by considering risk acceptability. Reliabil Eng Syst Safe. 2023;238:109446. doi: 10.1016/j.ress.2023.109446

[16] Santiago Oliveira S, de Albuquerque Soares W, Vasconcelos BM. Fatal fall-from-height accidents: statistical treatment using the human factors analysis and classification system – HFACS. J Safety Res. 2023;86:118–26. doi: 10.1016/j.jsr.2023.05.004 37718038

[17] Leveson NG. Engineering a Safer World: Systems Thinking Applied to Safety. The MIT Press. 2016. Available: https://library.oapen.org/handle/20.500.12657/26043.

[18] Wu Y, Fu G, Han M, Jia Q, Lyu Q, Wang Y, et al. Comparison of the theoretical elements and application characteristics of STAMP, FRAM, and 24Model: a major hazardous chemical explosion accident. J Loss Prevent Process Ind. 2022;80:104880. doi: 10.1016/j.jlp.2022.104880

[19] Ebrahimi H, Zarei E, Ansari M, Nojoumi A, Yarahmadi R. A system theory based accident analysis model: STAMP-fuzzy DEMATEL. Safe Sci. 2024;173:106445. doi: 10.1016/j.ssci.2024.106445

[20] Abedi M, Read GJM, McLean S, Wynne RA, Hulme A, Thompson J, et al. Causation and control: understanding distracted driving in Australia through a systems thinking lens. Safe Sci. 2024;173:106435. doi: 10.1016/j.ssci.2024.106435

[21] Meng H, An X, Li D, Zhao S, Zio E, Liu X, et al. A STAMP-Game model for accident analysis in oil and gas industry. Petroleum Sci. 2024;21(3):2154–67. doi: 10.1016/j.petsci.2023.12.002

[22] Borges SFDS, Albuquerque MAFD, Cardoso Junior MM, Belderrain MCN, Costa LELD. Systems Theoretic Process Analysis (STPA): a bibliometric and patents analysis. Gestão Produção. 2021;28:e5073.

[23] Maalouf MM, Hoque I. Applying fuzzy set qualitative comparative analysis to identify pathways for improving occupational health and safety performance. Safe Sci. 2022;156:105903. doi: 10.1016/j.ssci.2022.105903

[24] Schneider CQ, Wagemann C. Standards of good practice in qualitative comparative analysis (QCA) and fuzzy-sets. Comp Sociol. 2010;9:397–418.

[25] Ragin CC. The comparative method: Moving beyond qualitative and quantitative strategies. University of California Press. 2014. Available: https://books.google.com/books?hl=en&lr=&id=zLinAwAAQBAJ&oi=fnd&pg=PP1&dq=%5B14%5D%09Ragin,+C.+C.+(2014).+The+comparative+method:+Moving+beyond+qualitative+and+quantitative+strategies.+University+of+California+Press.&ots=_HkBqQr1d7&sig=gZAPyC49xmmMp8oCb7WUs3ThqYY.

[26] Vis B, Dul J. Analyzing relationships of necessity not just in kind but also in degree: complementing fsQCA with NCA. Sociol Methods Res. 2018;47(4):872–99. doi: 10.1177/0049124115626179 30443090 PMC6195096

[27] Dul J. Necessary Condition Analysis (NCA): Logic and Methodology of “Necessary but Not Sufficient” Causality. Organization Res Methods. 2015;19(1):10–52. doi: 10.1177/1094428115584005

[28] Niskanen T, Louhelainen K, Hirvonen ML. A systems thinking approach of occupational safety and health applied in the micro-, meso- and macro-levels: a Finnish survey. Safe Sci. 2016;82:212–27. doi: 10.1016/j.ssci.2015.09.012

[29] Leveson NG, Thomas JP. STPA Handbook. Cambridge, MA. 2018.

[30] Trist EL, Bamforth KW. Some social and psychological consequences of the Longwall method of coal-getting: an examination of the psychological situation and defences of a work group in relation to the social structure and technological content of the work system. Hum Relat. 1951;4(1):3–38. doi: 10.1177/001872675100400101

[31] Ashby WR. An Introduction to Cybernetics. London: Chapman & Hall; 1956.

[32] Read GJM, Naweed A, Salmon PM. Complexity on the rails: a systems-based approach to understanding safety management in rail transport. Reliabil Eng Syst Safe. 2019;188:352–65. doi: 10.1016/j.ress.2019.03.038

[33] Forrester JW. Industrial dynamics—after the first decade. Manage Sci. 1968;14(7):398–415. doi: 10.1287/mnsc.14.7.398

[34] Leveson N. A new accident model for engineering safer systems. Safe Sci. 2004;42(4):237–70. doi: 10.1016/s0925-7535(03)00047-x

[35] Ge J, Zhang Y, Xu K, Li J, Yao X, Wu C, et al. A new accident causation theory based on systems thinking and its systemic accident analysis method of work systems. Process Safe Environ Protect. 2022;158:644–60. doi: 10.1016/j.psep.2021.12.036

[36] Liu J, Dong C, An S. Integration and modularization: Research on urban cross-regional emergency cooperation based on the network approach. Int J Disaster Risk Reduct. 2022;82:103375. doi: 10.1016/j.ijdrr.2022.103375

[37] Del Sarto N, Isabelle DA, Di Minin A. The role of accelerators in firm survival: an fsQCA analysis of Italian startups. Technovation. 2020;90–91:102102. doi: 10.1016/j.technovation.2019.102102

[38] Giglio C, Popescu IA, Verteramo S. Do prosumers behave differently from other consumers on collaborative consumption platforms? Manag Decis. 2023; ahead-of-print. doi: 10.1108/MD-04-2023-0664

[39] Kumar S, Sahoo S, Ali F, Cobanoglu C. Rise of fsQCA in tourism and hospitality research: a systematic literature review. Int J Contemp Hosp Manag. 2023;36(7):2165–93. doi: 10.1108/ijchm-03-2023-0288

[40] Cervelló-Royo R, Moya-Clemente I, Perelló-Marín MR, Ribes-Giner G. Sustainable development, economic and financial factors, that influence the opportunity-driven entrepreneurship. An fsQCA approach. J Bus Res. 2020;115:393–402.

[41] Greckhamer T, Gur FA. Disentangling combinations and contingencies of generic strategies: a set-theoretic configurational approach. Long Range Plann. 2021;54(2):101951. doi: 10.1016/j.lrp.2019.101951

[42] Crilly D, Zollo M, Hansen MT. Faking it or muddling through? Understanding decoupling in response to stakeholder pressures. AMJ. 2012;55(6):1429–48. doi: 10.5465/amj.2010.0697

[43] Schneider MR, Schulze-Bentrop C, Paunescu M. Mapping the institutional capital of high-tech firms: a fuzzy-set analysis of capitalist variety and export performance. J Int Bus Stud. 2009;41(2):246–66. doi: 10.1057/jibs.2009.36

[44] Greckhamer T, Furnari S, Fiss PC, Aguilera RV. Studying configurations with qualitative comparative analysis: best practices in strategy and organization research. Strategic Organization. 2018;16:482–95.

[45] Gonçalves HM, Lourenço TF, Silva GM. Green buying behavior and the theory of consumption values: a fuzzy-set approach. J Bus Res. 2016;69:1484–91.

[46] Dul J, van der Laan E, Kuik R. A statistical significance test for necessary condition analysis. Organization Res Methods. 2018;23(2):385–95. doi: 10.1177/1094428118795272

[47] Fiss PC. Building better causal theories: a fuzzy set approach to typologies in organization research. AMJ. 2011;54(2):393–420. doi: 10.5465/amj.2011.60263120

[48] Hinsberg KL, Lamanna AJ. Crisis communication in construction: organizational strategies for worksite fatalities. J Safety Res. 2024;88:145–60. doi: 10.1016/j.jsr.2023.11.002 38485357

[49] Wang X, Yang Y, Chan APC, Chi H, Yung EHK. A regulatory framework for the use of small unmanned aircrafts (SUAs) in the construction industry. Eng Construct Archit Manage. 2023; ahead-of-print. doi: 10.1108/ECAM-10-2022-0990

[50] Gao S, Low SP, Howe HJA. Systemic lapses as the main causes of accidents in the Singapore construction industry. Civil Eng Environ Syst. 2018;35(1–4):81–98. doi: 10.1080/10286608.2018.1518437

[51] Singh SK. Road traffic accidents in India: issues and challenges. Transport Res Proc. 2017;25:4708–19. doi: 10.1016/j.trpro.2017.05.484

[52] Salmon PM, Read GJM, Stevens NJ. Who is in control of road safety? A STAMP control structure analysis of the road transport system in Queensland, Australia. Accid Anal Prev. 2016;96:140–51. doi: 10.1016/j.aap.2016.05.025 27526203

[53] Qiao W, Chen X, Xia W. STAMP-based causal analysis of the Coal Mine Major Accident: from the perspective of safety process. Energy Rep. 2021;7:116–24.

[54] Qiu Z, Liu Q, Li X, Zhang J, Zhang Y. Construction and analysis of a coal mine accident causation network based on text mining. Process Safe Environ Protect. 2021;153:320–8. doi: 10.1016/j.psep.2021.07.032

[55] Dash AK, Bhattacharjee RM, Paul PS. Lessons learnt from Indian inundation disasters: an analysis of case studies. Int J Disaster Risk Reduct. 2016;20:93–102. doi: 10.1016/j.ijdrr.2016.10.013

[56] Liu Q, Shang J, Wang J, Li M, Li T. Research on the evaluation of the operating effectiveness of the safety double prevention mechanism of coal mine enterprises based on matter-element extension. Process Safe Environ Protect. 2024;185:899–909. doi: 10.1016/j.psep.2024.03.073

[57] Kodur V, Kumar P, Rafi MM. Fire hazard in buildings: review, assessment and strategies for improving fire safety. PSU Res Rev. 2020;4:1–23.

[58] Kim JS, Kim BS. Analysis of fire-accident factors using big-data analysis method for construction areas. KSCE J Civil Eng. 2018;22:1535–43.

[59] Sedlar N, Irwin A, Martin D, Roberts R. A qualitative systematic review on the application of the normalization of deviance phenomenon within high-risk industries. J Safety Res. 2023;84:290–305. doi: 10.1016/j.jsr.2022.11.005 36868658

[60] Mallari CBC, San Juan JL, Chiu SF, Mayol AP, Yeo ES, Bacosa HP, et al. Modeling oil spill disasters using system dynamics: a case study on the MT Princess Empress oil spill in Oriental Mindoro, Philippines. Int J Disaster Risk Reduct. 2024;108:104524. doi: 10.1016/j.ijdrr.2024.104524

[61] Zhang Y, Wang W, Mi L, Liu Y, Qiao L, Ni G, et al. Deconstructing the organizational resilience of construction firms in major emergencies: a text mining analysis of listed construction companies in China. Int J Disaster Risk Reduct. 2024;106:104473. doi: 10.1016/j.ijdrr.2024.104473

[62] Nævestad T-O, Storesund Hesjevoll I, Elvik R. How can regulatory authorities improve safety in organizations by influencing safety culture? A conceptual model of the relationships and a discussion of implications. Accid Anal Prev. 2021;159:106228. doi: 10.1016/j.aap.2021.106228 34147704

[63] Zarei E, Khan F, Abbassi R. A dynamic human-factor risk model to analyze safety in sociotechnical systems. Process Safe Environ Protect. 2022;164:479–98. doi: 10.1016/j.psep.2022.06.040

[64] Ammar SF. The frontstage-backstage of organizational identity and management control system: the tale of British Petroleum’s embarrassment in DWH. SAMPJ. 2023;15(2):265–98. doi: 10.1108/sampj-11-2022-0584

[65] Yuxin W, Gui F, Qian L, Jingru W, Yali W, Meng H, et al. Accident case-driven study on the causal modeling and prevention strategies of coal-mine gas-explosion accidents: a systematic analysis of coal-mine accidents in China. Resour Policy. 2024;88:104425. doi: 10.1016/j.resourpol.2023.104425

[66] Marino M, Cavallaro L, Castro E, Musumeci RE, Martignoni M, Roman F, et al. New frontiers in the risk assessment of ship collision. Ocean Eng. 2023;274:113999. doi: 10.1016/j.oceaneng.2023.113999

[67] Zhang L, Shao Z, Benitez J. How to leverage digital platforms in preventing industrial accidents: evidence from a multi-level empirical study. Product Operat Manag. 2025. doi: 10.1177/10591478241305263

[68] Wang X, Qu Z, Song X, Bai Q, Pan Z, Li H. Incorporating accident liability into crash risk analysis: a multidimensional risk source approach. Accid Anal Prev. 2021;153:106035. doi: 10.1016/j.aap.2021.106035 33607319

[69] Qiao W. Analysis and measurement of multifactor risk in underground coal mine accidents based on coupling theory. Reliabil Eng Syst Safe. 2021;208:107433. doi: 10.1016/j.ress.2021.107433

[70] Kutsch E, Siala H, Cantarelli C, Djabbarov I. Categorizing errors in high-reliability organizations: adaptive range and adaptive capacity in incident response. Risk Anal. 2025. doi: 10.1111/risa.70024 40121089 PMC12411123

[71] Khan RU, Yin J, Mustafa FS, Shi W. Factor assessment of hazardous cargo ship berthing accidents using an ordered logit regression model. Ocean Eng. 2023;284:115211. doi: 10.1016/j.oceaneng.2023.115211

[72] Hinsberg KL, Lamanna AJ. Crisis communication in construction: organizational strategies for worksite fatalities. J Safety Res. 2024;88:145–60. doi: 10.1016/j.jsr.2023.11.002 38485357

[73] González Dan JR, Arnaldos J, Darbra RM. Introduction of the human factor in the estimation of accident frequencies through fuzzy logic. Safe Sci. 2017;97:134–43. doi: 10.1016/j.ssci.2015.08.012

[74] Mohammadfam I, Mirzaei Aliabadi M, Soltanian AR, Mahdinia M. Modeling the causes-effect relationships among major accident predictors based on a fuzzy multi-criteria decision-making method. Work. 2020;67(2):313–21. doi: 10.3233/WOR-203281 33044212

[75] Rostamzadeh S, Abouhossein A, Chalak MH, Vosoughi S, Norouzi R. An integrated DEMATEL-ANP approach for identification and prioritization of factors affecting fall from height accidents in the construction industry. Int J Occup Saf Ergon. 2023;29(2):474–83. doi: 10.1080/10803548.2022.2052479 35272574

[76] Delikhoon M, Zarei E, Banda OV, Faridan M, Habibi E. Systems thinking accident analysis models: a systematic review for sustainable safety management. Sustainability. 2022;14(10):5869. doi: 10.3390/su14105869

[77] Bouraima MB, Gore A, Ayyildiz E, Yalcin S, Badi I, Kiptum CK, et al. Assessing of causes of accidents based on a novel integrated interval-valued Fermatean fuzzy methodology: towards a sustainable construction site. Neural Comput Applic. 2023;35(29):21725–50. doi: 10.1007/s00521-023-08948-5

