# Supplementary material for: A systemic approach to accident prevention: How control factors influence accident severity and losses across industries
Source: PLoS One. 2025 Jun 20;20(6):e0325393. doi: 10.1371/journal.pone.0325393 (PMC12180727; doi:10.1371/journal.pone.0325393)
Supplement: S1 Appendix — (DOCX) [file pone.0325393.s001.docx]

The framework for writing accident investigation reports in China is as follows. The article primarily uses sections 6 (Analysis of Accident Causes) and 7 (Main Problems in the Responsible Units) for research.

1. Cover

- Title of the Accident Investigation Report
- Compilation organization
- Compilation date

1. Table of Contents

- Titles and page numbers of all chapters and subchapters in the report

3. Introduction and Determination of Accident Nature

- Time, location, unit, type, casualties, and economic losses of the accident
- Establishment of the accident investigation team and the investigation method
- Preliminary determination of the nature of the accident

4. Basic Information of the Accident

- Overview of the accident unit and related units
- Safety management situation of the accident unit
- Course of the accident
- Situation at the accident scene
- Casualties and direct economic losses
- Other relevant information

5. Emergency Response and Evaluation

- Accident information reporting and response
- Onsite emergency response
- Medical treatment and aftermath handling
- Emergency response evaluation

6. Analysis of Accident Causes

- - Direct Cause Analysis

Analyze each factor in the accident cause chain in chronological order of the accident, examining the unsafe behaviors of the individuals involved or the unsafe conditions of the equipment, and their causal relationships and degrees of association with the accident.

- - Inspection, Testing, and Appraisal of the Accident

Cite conclusions from inspections, tests, and appraisals provided by qualified institutions, with relevant evidence and supporting data included as attachments.

- - Exclusion of Other Possible Factors

Exclude factors such as intentional human sabotage and sudden disaster events through onsite investigations, interviews, and analysis of accident site video footage, with a focus on ruling out other factors of public and media concern.

- - Indirect Cause Analysis

Focus on analyzing the issues in safety management within the accident company, related units, and regulatory departments that are related to the occurrence of the accident.

7. Main Problems in the Responsible Units

- - Accident Unit

Detailed description of the main problems in the accident unit, intermediary agencies, and other related units.

- - Regulatory Departments

Detailed description of the main problems in regulatory departments regarding administrative approval, supervision, law enforcement, safety production work arrangements, special rectifications, inspections, and other duties.

- Local Party and Government

Detailed description of the main problems in local party and government bodies regarding the implementation of the party and state’s safety production policies, laws, and regulations.

8. Suggestions for Handling Responsible Personnel and Units

- Responsibility Determination

Determination of the responsibilities of the personnel and units involved.

- Handling Suggestions

Suggestions for administrative penalties and other actions.

9. Major Lessons from the Accident

- Analysis of Factors in the Accident Cause Chain

Summarize the illegal and noncompliant actions of responsible units and individuals.

Extract lessons learned from the accident.

10. Accident Rectification and Preventive Measures

- Rectification Measures

Propose rectification measures based on the lessons and cause analysis of the accident.

- Preventive Measures

Include improvements in policies, technology, and management.

11. Attachments

- List of accident investigation team members and signatures.
- Technical investigation report and management investigation report.
- Emergency response evaluation report.
- Technical appraisal analysis report from relevant units.
- Onsite investigation report.
- Other relevant materials.
