# Supplementary material for: A systemic approach to accident prevention: How control factors influence accident severity and losses across industries
Source: PLoS One. 2025 Jun 20;20(6):e0325393. doi: 10.1371/journal.pone.0325393 (PMC12180727; doi:10.1371/journal.pone.0325393)
Supplement: S2 Dataset — (DOCX) [file pone.0325393.s002.docx]

**Table : Database of Moderate, Major, and Extraordinarily Major Industrial Accidents in China by Industry Category (2011-2022)**

| **No.** | **Accident** | **Date** | **Deaths** | **Injuries** | **Direct Economic Loss (10,000 Yuan)** | **Accident Level** | **Industry** |
| --- | --- | --- | --- | --- | --- | --- | --- |
| 1 | Dalian Kunma Gas Co., Ltd. "9.10" Moderate LPG Pipeline Leakage and Explosion Accident | 2021.9.10 | 9 | 4 | 1797.5 | Moderate | Chemical |
| 2 | Suihua Anda City Bell Chemical Co., Ltd. "12.19" Moderate Emulsification Kettle Explosion Accident | 2020.12.19 | 3 | 4 | 2405 | Moderate | Chemical |
| 3 | Xiantao City Lanhua Organic Silicon Co., Ltd. "8.3" Moderate Explosion Accident | 2020.8.3 | 6 | 4 | 1344.18 | Moderate | Chemical |
| 4 | Zhangye City Gaotai County Zhangye Yaobang Chemical Technology Co., Ltd. "9.14" Moderate Poisoning Accident | 2020.9.14 | 3 | 0 | 450 | Moderate | Chemical |
| 5 | Suihua Anda City Heilongjiang Kailunda Technology Co., Ltd. "4.21" Moderate Poisoning and Asphyxiation Accident | 2021.4.21 | 4 | 9 | 873.3 | Moderate | Chemical |
| 6 | Tianmen City Chutian Biotechnology Co., Ltd. "9.28" Moderate Explosion Accident | 2020.9.28 | 6 | 1 | 542.5 | Moderate | Chemical |
| 7 | Inner Mongolia Yidong Group Dongxing Chemical "4.24" Moderate Production Safety Accident | 2019.4.24 | 4 | 36 | 4154 | Moderate | Chemical |
| 8 | Zhangjiakou Zhuolu County National Highway 109 "12.31" Road Traffic Accident | 2020.12.31 | 5 | 4 | 850 | Moderate | Transportation |
| 9 | Daqing Zhaozhou County Zhongya Petroleum Co., Ltd. "2.4" Moderate Vehicle-Mounted Tank Maintenance Explosion Accident | 2021.2.4 | 3 | 0 | 570.25 | Moderate | Transportation |
| 10 | National Highway 515 Tangyin Section "3.27" Moderate Road Traffic Accident | 2021.3.27 | 6 | 6 | 500 | Moderate | Transportation |
| 11 | Cangsuo Expressway "11.15" Moderate Road Traffic Accident | 2020.11.15 | 5 | 2 | 21.57 | Moderate | Transportation |
| 12 | Metro Line 4 Dengzhou South Road Station "10.12" Moderate Collapse Accident | 2021.10.12 | 4 | 1 | 667.626384 | Moderate | Construction |
| 13 | Ningxia Tianyuan Construction Co., Ltd. Construction Site "8.09" Collapse Accident | 2021.8.9 | 5 | 4 | 680 | Moderate | Construction |
| 14 | Jinchengjiang "Jinyi Times" Construction "2.8" Moderate Accident | 2018.2.8 | 3 | 1 | 280 | Moderate | Construction |
| 15 | Siming District Taiwan Mountain Villa "10.22" Moderate Villa Renovation Collapse Accident | 2020.10.22 | 3 | 0 | 438 | Moderate | Construction |
| 16 | Dongguan City Machong Town "6.7" Moderate Fall from Height Accident | 2015.6.7 | 4 | 0 | 450 | Moderate | Construction |
| 17 | Luhe County "10.8" Moderate Construction Accident | 2020.10.8 | 8 | 1 | 1163 | Moderate | Construction |
| 18 | Zhangjiakou City Chicheng County Maoyuan Mining Co., Ltd. "4.3" Moderate Roof Collapse and Concealment Accident | 2021.4.3 | 4 | 0 | 1000 | Moderate | Mining |
| 19 | Wuan City Metallurgical Mining Group Tuancheng East Mining Co., Ltd. "2.24" Moderate Mine Shaft Fall from Height Concealment Accident | 2021.2.24 | 6 | 0 | 1345 | Moderate | Mining |
| 20 | Xintai City Yangquan Mining Co., Ltd. "6.5" Moderate Roof Accident | 2022.6.5 | 3 | 1 | 509.2 | Moderate | Mining |
| 21 | Guizhou Liming Energy Group Co., Ltd. Jinsha County Xiluo Township Dongfeng Coal Mine "4.9" Moderate Coal and Gas Outburst Accident | 2021.4.9 | 8 | 1 | 1238.22 | Moderate | Mining |
| 22 | Qingyuan City Qingcheng District "2.16" Moderate Fire Accident | 2018.2.16 | 9 | 1 | 1200 | Moderate | Fire Protection |
| 23 | Zhijiang County Biyong Town Resident Self-built House "1.11" Moderate Fire Accident | 2022.1.11 | 5 | 1 | 76.88 | Moderate | Fire Protection |
| 24 | Bengbu City Longzihu District "11.15" Moderate Fire Accident | 2019.11.15 | 5 | 3 | 452.2 | Moderate | Fire Protection |
| 25 | Fuyang City Yingzhou District "6.22" Moderate Fire Accident | 2021.6.22 | 4 | 1 | 37.73 | Moderate | Fire Protection |
| 26 | Zhangjiakou China Chemical Group Shenghua Chemical Company "11.28" Major Explosion Accident | 2018.11.28 | 24 | 21 | 4148.8606 | Major | Chemical |
| 27 | Yibin Hengda Technology Co., Ltd. "7.12" Major Explosion and Fire Accident | 2018.7.12 | 19 | 12 | 4142 | Major | Chemical |
| 28 | Jiangsu Lianyungang Juxin Biotechnology Co., Ltd. "12.9" Major Explosion Accident | 2017.12.9 | 10 | 1 | 4875 | Major | Chemical |
| 29 | Shandong Linyi Jinyu Petrochemical Co., Ltd. "6.5" Tank Truck Leakage Major Explosion and Fire Accident | 2017.6.5 | 10 | 9 | 4468 | Major | Chemical |
| 30 | Dongying Shandong Binyuan Chemical Co., Ltd. "8.31" Major Explosion Accident | 2015.8.31 | 13 | 25 | 4326 | Major | Chemical |
| 31 | Boxing County Chengli Gas Supply Co., Ltd. "10.8" Major Explosion Accident | 2013.10.8 | 10 | 33 | 3200 | Major | Chemical |
| 32 | Shijiazhuang City Pingshan County Gangcheng Road Hutuo River Section "10.11" Vehicle Water Fall Major Accident | 2021.10.11 | 14 | 0 | 3500 | Major | Transportation |
| 33 | Songyuan City "10.4" Major Road Traffic Accident | 2020.10.4 | 18 | 1 | 606.1 | Major | Transportation |
| 34 | Xi'an "11.13" Major Road Traffic Accident | 2018.11.13 | 10 | 2 | 635 | Major | Transportation |
| 35 | Guanghe Expressway Longmen Section "7.6" Major Road Traffic Accident | 2017.7.6 | 19 | 31 | 3152.17 | Major | Transportation |
| 36 | Guangzhou "6.29" Road Traffic Accident Leading to Major Explosion Accident | 2012.6.29 | 20 | 31 | 4600 | Major | Transportation |
| 37 | Hebei Baoding Zhangshi Expressway Futuyu No. 5 Tunnel "5.23" Major Hazardous Chemical Transport Vehicle Combustion and Explosion Accident | 2017.5.23 | 15 | 19 | 4200 | Major | Transportation |
| 38 | Beijing-Hong Kong-Macao Expressway Hengyang Section "6.29" Major Road Traffic Accident | 2018.6.29 | 18 | 14 | 2632.8 | Major | Transportation |
| 39 | Yuegan Expressway Heyuan Heping Section "12.13" Major Road Traffic Accident | 2014.12.13 | 12 | 3 | 1264.3 | Major | Transportation |
| 40 | Dalian City Lvshunkou District Lanwan Phase III Construction Site "10.8" Major Construction Collapse Accident | 2011.10.8 | 13 | 5 | 1237.72 | Major | Construction |
| 41 | Fujian Province Quanzhou City Xinjia Hotel "3.7" Collapse Accident | 2020.3.7 | 29 | 42 | 5794 | Major | Construction |
| 42 | Hengshui City Feicui Huating "4.25" Construction Elevator Car Fall Major Accident | 2019.4.25 | 11 | 2 | 1800 | Major | Construction |
| 43 | Tsinghua University Affiliated Middle School Gymnasium and Dormitory Project "11.28" Raft Foundation Reinforcement System Collapse Major Production Accident | 2014.12.29 | 10 | 4 | 0 | Major | Construction |
| 44 | Guangdong Province Foshan City Rail Transit Line 2 Phase I Project "2.7" Water Inrush Collapse Major Accident | 2018.2.7 | 12 | 8 | 5323.8 | Major | Construction |
| 45 | Linyi Lanling County Lanling Shuntian Transport Co., Ltd. "5.9" Retaining Wall Major Collapse Accident | 2015.5.9 | 10 | 3 | 721.5 | Major | Construction |
| 46 | Gaozhou City Shenzhen Town Under-construction Stone Arch Bridge "5.3" Major Collapse Accident | 2014.5.3 | 11 | 16 | 1015.6 | Major | Construction |
| 47 | Hubei Province Donghu Scenery Garden Construction Site "9.13" Major Construction Elevator Fall Accident | 2012.9.13 | 19 | 0 | 1800 | Major | Construction |
| 48 | Guizhou Province Qianxinan Prefecture Anlong County Guanglong Coal Mine "12.16" Major Coal and Gas Outburst Accident | 2019.12.16 | 16 | 1 | 2311 | Major | Mining |
| 49 | Shaanxi Province Yulin City Shenmu City Baiji Mining Co., Ltd. "1.12" Major Coal Dust Explosion Accident | 2019.1.12 | 21 | 0 | 3788 | Major | Mining |
| 50 | Hunan Province Hengyang City Leiyang City Daozi Coal Industry Co., Ltd. Yuanjiangshan Coal Mine "11.29" Major Water Inrush Accident | 2020.11.29 | 13 | 0 | 3484.03 | Major | Mining |
| 51 | Chongqing City Yongchuan District Diaoshuidong Coal Industry Co., Ltd. "12.4" Major Fire Accident | 2020.12.4 | 23 | 1 | 2632 | Major | Mining |
| 52 | Shandong Longyun Coal Industry Co., Ltd. "10.20" Major Rock Burst Accident | 2018.10.20 | 21 | 4 | 5639.8 | Major | Mining |
| 53 | Hunan Province Loudi City Lianyuan City Zubao Coal Mine "2.14" Runaway Vehicle Leading to Major Coal Dust Accident | 2017.2.14 | 10 | 2 | 2015 | Major | Mining |
| 54 | Shanxi Province Tongchuan City Zhaojin Mining Co., Ltd. "4.25" Major Water Hazard Accident | 2016.4.25 | 11 | 0 | 1838.17 | Major | Mining |
| 55 | Chongqing Energy Investment Yuxin Energy Co., Ltd. Songzao Coal Mine "9.27" Major Fire Accident | 2020.9.27 | 16 | 42 | 2501 | Major | Mining |
| 56 | Guizhou Sanhe Shunxun Coal Mine "2.25" Major Roof Accident | 2022.2.25 | 14 | 0 | 2288.47 | Major | Mining |
| 57 | Xinjiang Changji Prefecture Hutubi County Baiyangou Fengyuan Coal Mine "4.10" Major Water Inrush Accident | 2021.4.10 | 21 | 0 | 7067.2 | Major | Mining |
| 58 | Guangdong Province Foshan City Sanshui District Shengfeng Ceramics Co., Ltd. "8.23" Major Fire Accident | 2011.8.23 | 15 | 1 | 1113 | Major | Fire Protection |
| 59 | Harbin Beilong Hot Spring Leisure Hotel Co., Ltd. "8.25" Major Fire Accident | 2018.8.25 | 20 | 23 | 2504.8 | Major | Fire Protection |
| 60 | Tianjin City Hexi District Junyi Building No. 1 "12.1" Major Fire | 2017.12.1 | 10 | 5 | 2516.6 | Major | Fire Protection |
| 61 | Daxing District "11.18" Major Accident | 2017.11.18 | 19 | 8 | 0 | Major | Fire Protection |
| 62 | Weifang City Shouguang City Longyuan Food Co., Ltd. "11.16" Major Fire Accident | 2014.11.16 | 18 | 13 | 2666.2 | Major | Fire Protection |
| 63 | Jieyang City "3.26" Major Fire Accident | 2014.3.26 | 12 | 5 | 390.93 | Major | Fire Protection |
| 64 | Taizhou Dadong Shoe Industry Co., Ltd. "1.14" Major Fire Accident | 2014.1.14 | 16 | 5 | 0 | Major | Fire Protection |
| 65 | Shenzhen City "12.11" Major Fire Accident | 2013.12.11 | 16 | 5 | 1781.2 | Major | Fire Protection |
| 66 | Binhai New Area Zhongtang Town Sinotrans Jiuling Storage Warehouse "10.28" Major Fire Accident | 2018.10.28 | 0 | 0 | 8944.95 | Major | Fire Protection |
| 67 | Henan Province Zhecheng County "6.25" Major Fire Accident | 2021.6.25 | 18 | 11 | 2153.7 | Major | Fire Protection |
| 68 | Jiangsu Xiangshui Tianjiayi Chemical Co., Ltd. "3.21" Extraordinarily Major Explosion Accident | 2019.3.21 | 78 | 76 | 198635.07 | Extraordinarily Major | Chemical |
| 69 | Tianjin Port "8.12" Ruihai Company Dangerous Goods Warehouse Extraordinarily Major Fire and Explosion Accident | 2015.8.12 | 165 | 798 | 686600 | Extraordinarily Major | Chemical |
| 70 | Shandong Province Qingdao City "11.22" Sinopec East-Yellow Oil Pipeline Leakage Explosion Extraordinarily Major Accident | 2013.11.22 | 62 | 136 | 75172 | Extraordinarily Major | Chemical |
| 71 | Jingji Expressway Shanxi Jincheng Section Yanhou Tunnel "3.1" Extraordinarily Major Road Traffic Hazardous Chemical Combustion and Explosion Accident | 2014.3.1 | 40 | 12 | 8197 | Extraordinarily Major | Transportation |
| 72 | Changshen Expressway Jiangsu Wuxi "9.28" Extraordinarily Major Road Traffic Accident | 2019.9.28 | 36 | 36 | 7100 | Extraordinarily Major | Transportation |
| 73 | Shaanxi Ankang Jingkun Expressway "8.10" Extraordinarily Major Road Traffic Accident | 2017.8.10 | 36 | 13 | 3533 | Extraordinarily Major | Transportation |
| 74 | Hunan Chenzhou Yifeng Expressway "6.26" Extraordinarily Major Road Traffic Accident | 2016.6.26 | 35 | 13 | 2290 | Extraordinarily Major | Transportation |
| 75 | Jiangxi Fengcheng Power Plant "11.24" Cooling Tower Construction Platform Collapse Extraordinarily Major Accident | 2016.11.24 | 73 | 2 | 10197.2 | Extraordinarily Major | Construction |
| 76 | Guangdong Shenzhen Guangming New District Earth Dump Site "12.20" Extraordinarily Major Landslide Accident | 2015.12.20 | 73 | 17 | 8812.23 | Extraordinarily Major | Construction |
| 77 | Chongqing City Yongchuan District Jinshanggou Coal Industry Co., Ltd. "10.31" Extraordinarily Major Gas Explosion Accident | 2016.10.31 | 33 | 1 | 3682.22 | Extraordinarily Major | Mining |
| 78 | Inner Mongolia Autonomous Region Chifeng Baoma Mining Co., Ltd. "12.3" Extraordinarily Major Gas Explosion Accident | 2016.12.3 | 32 | 20 | 4399 | Extraordinarily Major | Mining |
| 79 | Jilin Province Changchun City Baoyuanfeng Poultry Industry Co., Ltd. "6.3" Extraordinarily Major Fire and Explosion Accident | 2013.6.3 | 121 | 76 | 18200 | Extraordinarily Major | Fire Protection |
| 80 | Henan Pingdingshan "5.25" Extraordinarily Major Fire Accident | 2015.5.25 | 39 | 6 | 2064.5 | Extraordinarily Major | Fire Protection |

**Table : NCA Results for the Five Major Industry Sectors**

| **Industry** | **Outcome Variable** | **Control System Component** | **Method** | **Accuracy** | **Ceiling Zone** | **Scope** | **Effect Size (d)** | **p-value** |
| --- | --- | --- | --- | --- | --- | --- | --- | --- |
| **Chemical Industry** | Death toll | Control activities | CR | 75.0% | 0.300 | 0.87 | 0.343 | 0.004 |
|  |  |  | CE | 100.0% | 0.311 | 0.87 | 0.356 | 0.010 |
|  |  | Feedback | CR | 93.8% | 0.185 | 0.88 | 0.209 | 0.003 |
|  |  |  | CE | 100.0% | 0.293 | 0.88 | 0.332 | 0.000 |
|  |  | Controller failure | CR | 81.2% | 0.278 | 0.87 | 0.318 | 0.026 |
|  |  |  | CE | 100.0% | 0.324 | 0.87 | 0.151 | 0.267 |
|  |  | Controlled process | CR | 100.0% | 0.066 | 0.87 | 0.075 | 0.311 |
|  |  |  | CE | 100.0% | 0.132 | 0.87 | 0.151 | 0.267 |
|  | Injuries | Control activities | CR | 87.5% | 0.214 | 0.89 | 0.240 | 0.153 |
|  |  |  | CE | 100.0% | 0.321 | 0.89 | 0.360 | 0.056 |
|  |  | Feedback | CR | 87.5% | 0.205 | 0.90 | 0.227 | 0.030 |
|  |  |  | CE | 100.0% | 0.289 | 0.90 | 0.320 | 0.045 |
|  |  | Controller failure | CR | 87.5% | 0.213 | 0.89 | 0.239 | 0.230 |
|  |  |  | CE | 100.0% | 0.313 | 0.89 | 0.350 | 0.125 |
|  |  | Controlled process | CR | 100.0% | 0.106 | 0.89 | 0.118 | 0.446 |
|  |  |  | CE | 100.0% | 0.211 | 0.89 | 0.237 | 0.428 |
|  | Economic loss | Control activities | CR | 87.5% | 0.300 | 0.88 | 0.340 | 0.023 |
|  |  |  | CE | 100.0% | 0.383 | 0.88 | 0.433 | 0.018 |
|  |  | Feedback | CR | 93.8% | 0.170 | 0.89 | 0.190 | 0.045 |
|  |  |  | CE | 100.0% | 0.264 | 0.89 | 0.296 | 0.047 |
|  |  | Controller failure | CR | 87.5% | 0.302 | 0.88 | 0.342 | 0.035 |
|  |  |  | CE | 100.0% | 0.365 | 0.88 | 0.414 | 0.051 |
|  |  | Controlled process | CR | 100.0% | 0.154 | 0.88 | 0.174 | 0.209 |
|  |  |  | CE | 100.0% | 0.308 | 0.88 | 0.349 | 0.139 |
| **Construction Industry** | Death toll | Control activities | CR | 81.2% | 0.321 | 0.84 | 0.384 | 0.001 |
|  |  |  | CE | 100.0% | 0.392 | 0.84 | 0.469 | 0.000 |
|  |  | Feedback | CR | 81.2% | 0.225 | 0.84 | 0.269 | 0.008 |
|  |  |  | CE | 100.0% | 0.327 | 0.84 | 0.390 | 0.010 |
|  |  | Controller failure | CR | 62.5% | 0.442 | 0.82 | 0.540 | 0.000 |
|  |  |  | CE | 100.0% | 0.510 | 0.82 | 0.623 | 0.000 |
|  |  | Controlled process | CR | 100.0% | 0.057 | 0.40 | 0.140 | 0.010 |
|  |  |  | CE | 100.0% | 0.113 | 0.40 | 0.280 | 0.006 |
|  | Injuries | Control activities | CR | 93.8% | 0.163 | 0.88 | 0.184 | 0.208 |
|  |  |  | CE | 100.0% | 0.236 | 0.88 | 0.268 | 0.069 |
|  |  | Feedback | CR | 87.5% | 0.203 | 0.88 | 0.230 | 0.029 |
|  |  |  | CE | 100.0% | 0.269 | 0.88 | 0.304 | 0.054 |
|  |  | Controller failure | CR | 87.5% | 0.297 | 0.86 | 0.344 | 0.010 |
|  |  |  | CE | 100.0% | 0.332 | 0.86 | 0.384 | 0.020 |
|  |  | Controlled process | CR | 87.5% | 0.044 | 0.42 | 0.103 | 0.026 |
|  |  |  | CE | 100.0% | 0.061 | 0.42 | 0.143 | 0.040 |
|  | Economic loss | Control activities | CR | 81.2% | 0.393 | 0.87 | 0.449 | 0.000 |
|  |  |  | CE | 100.0% | 0.436 | 0.87 | 0.499 | 0.000 |
|  |  | Feedback | CR | 87.5% | 0.302 | 0.87 | 0.345 | 0.000 |
|  |  |  | CE | 100.0% | 0.449 | 0.87 | 0.514 | 0.000 |
|  |  | Controller failure | CR | 56.2% | 0.437 | 0.86 | 0.511 | 0.000 |
|  |  |  | CE | 100.0% | 0.507 | 0.86 | 0.593 | 0.000 |
|  |  | Controlled process | CR | 93.8% | 0.056 | 0.423 | 0.134 | 0.003 |
|  |  |  | CE | 100.0% | 0.086 | 0.42 | 0.203 | 0.002 |
| **Transportation Industry** | Death toll | Control activities | CR | 87.5% | 0.313 | 0.87 | 0.358 | 0.000 |
|  |  |  | CE | 100.0% | 0.425 | 0.87 | 0.487 | 0.000 |
|  |  | Feedback | CR | 93.8% | 0.178 | 0.88 | 0.202 | 0.035 |
|  |  |  | CE | 100.0% | 0.214 | 0.88 | 0.243 | 0.051 |
|  |  | Controller failure | CR | 87.5% | 0.244 | 0.92 | 0.265 | 0.016 |
|  |  |  | CE | 100.0% | 0.200 | 0.92 | 0.217 | 0.047 |
|  |  | Controlled process | CR | 100.0% | 0.000 | 0.89 | 0.000 | 0.000 |
|  |  |  | CE | 100.0% | 0.000 | 0.89 | 0.000 | 1.000 |
|  | Injuries | Control activities | CR | 81.2% | 0.281 | 0.86 | 0.328 | 0.002 |
|  |  |  | CE | 100.0% | 0.339 | 0.86 | 0.396 | 0.002 |
|  |  | Feedback | CR | 87.5% | 0.255 | 0.86 | 0.295 | 0.003 |
|  |  |  | CE | 100.0% | 0.325 | 0.86 | 0.376 | 0.006 |
|  |  | Controller failure | CR | 81.2% | 0.220 | 0.90 | 0.244 | 0.047 |
|  |  |  | CE | 100.0% | 0.214 | 0.90 | 0.238 | 0.042 |
|  |  | Controlled process | CR | 93.8% | 0.062 | 0.87 | 0.071 | 0.211 |
|  |  |  | CE | 100.0% | 0.095 | 0.87 | 0.109 | 0.218 |
|  | Economic loss | Control activities | CR | 87.5% | 0.322 | 0.87 | 0.368 | 0.000 |
|  |  |  | CE | 100.0% | 0.442 | 0.87 | 0.505 | 0.000 |
|  |  | Feedback | CR | 93.8% | 0.224 | 0.88 | 0.254 | 0.022 |
|  |  |  | CE | 100.0% | 0.322 | 0.88 | 0.364 | 0.008 |
|  |  | Controller failure | CR | 87.5% | 0.265 | 0.92 | 0.288 | 0.016 |
|  |  |  | CE | 100.0% | 0.325 | 0.92 | 0.353 | 0.001 |
|  |  | Controlled process | CR | 100.0% | 0.000 | 0.89 | 0.000 | 1.000 |
|  |  |  | CE | 100.0% | 0.000 | 0.89 | 0.000 | 1.000 |
| **Coal Mining Industry** | Death toll | Control activities | CR | 87.5% | 0.205 | 0.87 | 0.234 | 0.021 |
|  |  |  | CE | 100.0% | 0.193 | 0.87 | 0.221 | 0.015 |
|  |  | Feedback | CR | 100.0% | 0.036 | 0.83 | 0.043 | 0.542 |
|  |  |  | CE | 100.0% | 0.072 | 0.83 | 0.087 | 0.542 |
|  |  | Controller failure | CR | 81.2% | 0.271 | 0.86 | 0.317 | 0.008 |
|  |  |  | CE | 100.0% | 0.363 | 0.86 | 0.425 | 0.000 |
|  |  | Controlled process | CR | 87.5% | 0.109 | 0.86 | 0.127 | 0.016 |
|  |  |  | CE | 100.0% | 0.161 | 0.86 | 0.187 | 0.033 |
|  | Injuries | Control activities | CR | 75.0% | 0.277 | 0.89 | 0.310 | 0.031 |
|  |  |  | CE | 100.0% | 0.304 | 0.89 | 0.340 | 0.030 |
|  |  | Feedback | CR | 100.0% | 0.090 | 0.85 | 0.106 | 0.541 |
|  |  |  | CE | 100.0% | 0.180 | 0.85 | 0.213 | 0.541 |
|  |  | Controller failure | CR | 81.2% | 0.241 | 0.87 | 0.276 | 0.131 |
|  |  |  | CE | 100.0% | 0.342 | 0.87 | 0.391 | 0.099 |
|  |  | Controlled process | CR | 100.0% | 0.104 | 0.88 | 0.117 | 0.266 |
|  |  |  | CE | 100.0% | 0.207 | 0.88 | 0.234 | 0.254 |
|  | Economic loss | Control activities | CR | 100.0% | 0.235 | 0.64 | 0.369 | 0.003 |
|  |  |  | CE | 100.0% | 0.046 | 0.91 | 0.050 | 0.549 |
|  |  | Feedback | CR | 100.0% | 0.011 | 0.86 | 0.013 | 0.744 |
|  |  |  | CE | 100.0% | 0.023 | 0.86 | 0.026 | 0.744 |
|  |  | Controller failure | CR | 81.2% | 0.158 | 0.89 | 0.177 | 0.180 |
|  |  |  | CE | 100.0% | 0.246 | 0.89 | 0.242 | 0.038 |
|  |  | Controlled process | CR | 100.0% | 0.000 | 0.90 | 0.000 | 1.000 |
|  |  |  | CE | 100.0% | 0.000 | 0.90 | 0.000 | 1.000 |
| **Fire Fighting Industry** | Death toll | Control activities | CR | 68.8% | 0.505 | 0.93 | 0.542 | 0.000 |
|  |  |  | CE | 100.0% | 0.564 | 0.93 | 0.605 | 0.000 |
|  |  | Feedback | CR | 100.0% | 0.110 | 0.92 | 0.120 | 0.042 |
|  |  |  | CE | 100.0% | 0.221 | 0.92 | 0.240 | 0.042 |
|  |  | Controller failure | CR | 75.0% | 0.480 | 0.93 | 0.516 | 0.000 |
|  |  |  | CE | 100.0% | 0.555 | 0.93 | 0.596 | 0.000 |
|  |  | Controlled process | CR | 100.0% | 0.000 | 0.43 | 0.000 | 1.000 |
|  |  |  | CE | 100.0% | 0.000 | 0.43 | 0.000 | 1.000 |
|  | Injuries | Control activities | CR | 87.5% | 0.422 | 0.93 | 0.453 | 0.000 |
|  |  |  | CE | 100.0% | 0.500 | 0.93 | 0.537 | 0.000 |
|  |  | Feedback | CR | 100.0% | 0.113 | 0.92 | 0.122 | 0.040 |
|  |  |  | CE | 100.0% | 0.225 | 0.92 | 0.244 | 0.040 |
|  |  | Controller failure | CR | 75.0% | 0.368 | 0.93 | 0.395 | 0.001 |
|  |  |  | CE | 100.0% | 0.432 | 0.93 | 0.464 | 0.000 |
|  |  | Controlled process | CR | 100.0% | 0.000 | 0.43 | 0.000 | 1.000 |
|  |  |  | CE | 100.0% | 0.000 | 0.43 | 0.000 | 1.000 |
|  | Economic loss | Control activities | CR | 100.0% | 0.043 | 0.90 | 0.048 | 0.690 |
|  |  |  | CE | 100.0% | 0.086 | 0.90 | 0.096 | 0.010 |
|  |  | Feedback | CR | 100.0% | 0.020 | 0.89 | 0.023 | 0.559 |
|  |  |  | CE | 100.0% | 0.041 | 0.89 | 0.045 | 0.559 |
|  |  | Controller failure | CR | 81.2% | 0.304 | 0.90 | 0.337 | 0.013 |
|  |  |  | CE | 100.0% | 0.281 | 0.90 | 0.311 | 0.105 |
|  |  | Controlled process | CR | 100.0% | 0.000 | 0.42 | 0.000 | 1.000 |
|  |  |  | CE | 100.0% | 0.000 | 0.42 | 0.000 | 1.000 |

**Note:** CR = Ceiling Regression; CE = Ceiling Envelopment

**Table: Comprehensive QCA Method Original Data for Five Industries**

| **Industry** | **Num** | **DT** | **IN** | **EL** | **Grade** | **CA** | **F** | **CF** | **CP** |
| --- | --- | --- | --- | --- | --- | --- | --- | --- | --- |
| **Chemical** | 1 | 9 | 4 | 1797.5 | 2 | 4 | 0 | 7 | 1 |
|  | 2 | 3 | 4 | 2405 | 2 | 3 | 0 | 11 | 1 |
|  | 3 | 6 | 4 | 1344.18 | 2 | 6 | 0 | 8 | 0 |
|  | 4 | 3 | 0 | 450 | 2 | 12 | 0 | 14 | 0 |
|  | 5 | 4 | 9 | 873.3 | 2 | 4 | 0 | 12 | 0 |
|  | 6 | 6 | 1 | 542.5 | 2 | 5 | 0 | 11 | 0 |
|  | 7 | 4 | 36 | 4154 | 2 | 2 | 0 | 5 | 1 |
|  | 8 | 24 | 21 | 4148.8606 | 3 | 8 | 1 | 12 | 2 |
|  | 9 | 19 | 12 | 4142 | 3 | 53 | 5 | 31 | 7 |
|  | 10 | 10 | 1 | 4875 | 3 | 21 | 1 | 19 | 5 |
|  | 11 | 10 | 9 | 4468 | 3 | 46 | 1 | 30 | 5 |
|  | 12 | 13 | 25 | 4326 | 3 | 13 | 1 | 21 | 5 |
|  | 13 | 10 | 33 | 3200 | 3 | 7 | 1 | 9 | 0 |
|  | 14 | 78 | 76 | 198635.07 | 4 | 42 | 1 | 23 | 5 |
|  | 15 | 165 | 798 | 686600 | 4 | 61 | 2 | 21 | 5 |
|  | 16 | 62 | 136 | 75172 | 4 | 12 | 3 | 17 | 0 |
| **Construction** | 1 | 4 | 1 | 667.626384 | 2 | 5 | 1 | 8 | 0 |
|  | 2 | 5 | 4 | 680 | 2 | 4 | 0 | 7 | 0 |
|  | 3 | 3 | 1 | 280 | 2 | 3 | 0 | 4 | 0 |
|  | 4 | 3 | 0 | 438 | 2 | 4 | 1 | 2 | 0 |
|  | 5 | 4 | 0 | 450 | 2 | 3 | 1 | 2 | 0 |
|  | 6 | 8 | 1 | 1163 | 2 | 13 | 1 | 13 | 0 |
|  | 7 | 13 | 5 | 1237.72 | 3 | 6 | 1 | 9 | 0 |
|  | 8 | 29 | 42 | 5794 | 3 | 30 | 4 | 25 | 5 |
|  | 9 | 11 | 2 | 1800 | 3 | 14 | 2 | 22 | 1 |
|  | 10 | 10 | 4 | 0 | 3 | 2 | 0 | 9 | 0 |
|  | 11 | 12 | 8 | 5323.8 | 3 | 11 | 3 | 21 | 1 |
|  | 12 | 10 | 3 | 721.5 | 3 | 7 | 0 | 8 | 0 |
|  | 13 | 11 | 16 | 1015.6 | 3 | 6 | 1 | 8 | 0 |
|  | 14 | 19 | 0 | 1800 | 3 | 15 | 1 | 15 | 5 |
|  | 15 | 73 | 2 | 10197.2 | 4 | 40 | 2 | 30 | 2 |
|  | 16 | 73 | 17 | 8812.23 | 4 | 38 | 3 | 33 | 3 |
| **Transportation** | 1 | 5 | 4 | 850 | 2 | 8 | 0 | 2 | 9 |
|  | 2 | 3 | 0 | 570.25 | 2 | 3 | 0 | 8 | 1 |
|  | 3 | 6 | 6 | 500 | 2 | 5 | 1 | 6 | 0 |
|  | 4 | 5 | 2 | 21.57 | 2 | 4 | 1 | 8 | 1 |
|  | 5 | 14 | 0 | 3500 | 3 | 12 | 1 | 13 | 0 |
|  | 6 | 18 | 1 | 606.1 | 3 | 17 | 1 | 36 | 2 |
|  | 7 | 10 | 2 | 635 | 3 | 3 | 1 | 9 | 1 |
|  | 8 | 19 | 31 | 3152.17 | 3 | 14 | 5 | 20 | 2 |
|  | 9 | 20 | 31 | 4600 | 3 | 10 | 2 | 13 | 1 |
|  | 10 | 15 | 19 | 4200 | 3 | 10 | 2 | 15 | 0 |
|  | 11 | 18 | 14 | 2632.8 | 3 | 10 | 2 | 8 | 3 |
|  | 12 | 12 | 3 | 1264.3 | 3 | 6 | 2 | 15 | 2 |
|  | 13 | 40 | 12 | 8197 | 4 | 17 | 2 | 19 | 0 |
|  | 14 | 36 | 36 | 7100 | 4 | 21 | 3 | 19 | 3 |
|  | 15 | 36 | 13 | 3533 | 4 | 27 | 2 | 16 | 1 |
|  | 16 | 35 | 13 | 2290 | 4 | 15 | 1 | 11 | 0 |
| **Coal Mining** | 1 | 4 | 0 | 1000 | 2 | 5 | 2 | 12 | 1 |
|  | 2 | 6 | 0 | 1345 | 2 | 6 | 1 | 10 | 0 |
|  | 3 | 3 | 1 | 509.2 | 2 | 6 | 1 | 11 | 0 |
|  | 4 | 8 | 1 | 1238.22 | 2 | 6 | 1 | 14 | 1 |
|  | 5 | 16 | 1 | 2311 | 3 | 8 | 1 | 12 | 0 |
|  | 6 | 21 | 0 | 3788 | 3 | 9 | 1 | 16 | 1 |
|  | 7 | 13 | 0 | 3484.03 | 3 | 8 | 1 | 8 | 1 |
|  | 8 | 23 | 1 | 2632 | 3 | 7 | 0 | 21 | 1 |
|  | 9 | 21 | 4 | 5639.8 | 3 | 12 | 0 | 18 | 1 |
|  | 10 | 10 | 2 | 2015 | 3 | 8 | 1 | 11 | 0 |
|  | 11 | 11 | 0 | 1838.17 | 3 | 6 | 0 | 12 | 0 |
|  | 12 | 16 | 42 | 2501 | 3 | 11 | 1 | 16 | 1 |
|  | 13 | 14 | 0 | 2288.47 | 3 | 12 | 0 | 15 | 2 |
|  | 14 | 21 | 0 | 7067.2 | 3 | 6 | 1 | 20 | 0 |
|  | 15 | 33 | 1 | 3682.22 | 4 | 28 | 1 | 21 | 3 |
|  | 16 | 32 | 20 | 4399 | 4 | 34 | 2 | 21 | 6 |
| **Fire Fighting** | 1 | 9 | 1 | 1200 | 2 | 6 | 0 | 6 | 0 |
|  | 2 | 5 | 1 | 76.88 | 2 | 5 | 0 | 5 | 0 |
|  | 3 | 5 | 3 | 452.2 | 2 | 7 | 0 | 4 | 0 |
|  | 4 | 4 | 1 | 37.73 | 2 | 4 | 0 | 3 | 0 |
|  | 5 | 15 | 1 | 1113 | 3 | 9 | 1 | 9 | 0 |
|  | 6 | 20 | 23 | 2504.8 | 3 | 15 | 2 | 14 | 1 |
|  | 7 | 10 | 5 | 2516.6 | 3 | 9 | 1 | 8 | 0 |
|  | 8 | 19 | 8 | 0 | 3 | 14 | 1 | 14 | 1 |
|  | 9 | 18 | 13 | 2666.2 | 3 | 16 | 1 | 14 | 0 |
|  | 10 | 12 | 5 | 390.93 | 3 | 10 | 0 | 10 | 0 |
|  | 11 | 16 | 5 | 0 | 3 | 10 | 1 | 13 | 0 |
|  | 12 | 16 | 5 | 1781.2 | 3 | 12 | 0 | 14 | 1 |
|  | 13 | 0 | 0 | 8944.95 | 3 | 3 | 0 | 9 | 0 |
|  | 14 | 18 | 11 | 2153.7 | 3 | 10 | 1 | 10 | 0 |
|  | 15 | 121 | 76 | 18200 | 4 | 28 | 1 | 34 | 0 |
|  | 16 | 39 | 6 | 2064.5 | 4 | 20 | 1 | 22 | 1 |

**Legend:**

- **Num**: Case Number
- **DT**: Death Toll
- **IN**: Injuries
- **EL**: Economic Loss (10,000 Yuan)
- **Grade**: Accident Severity Grade (2=Moderate, 3=Major, 4=Extraordinarily Major)
- **CA**: Control Activities error
- **F**: Feedback error
- **CF**: Controller Failure
- **CP**: Controlled Process error
